# Barley Anther and Meiocyte Transcriptome Dynamics in Meiotic Prophase I

**DOI:** 10.3389/fpls.2020.619404

**Published:** 2021-01-12

**Authors:** Abdellah Barakate, Jamie Orr, Miriam Schreiber, Isabelle Colas, Dominika Lewandowska, Nicola McCallum, Malcolm Macaulay, Jenny Morris, Mikel Arrieta, Pete E. Hedley, Luke Ramsay, Robbie Waugh

**Affiliations:** ^1^Cell and Molecular Sciences, The James Hutton Institute, Dundee, United Kingdom; ^2^School of Life Sciences, University of Dundee, Dundee, United Kingdom; ^3^School of Agriculture and Wine, University of Adelaide, Adelaide, SA, Australia

**Keywords:** barley, anther, meiocyte, transcriptome, meiosis, argonaute, lncRNAs, ubiquitin

## Abstract

In flowering plants, successful germinal cell development and meiotic recombination depend upon a combination of environmental and genetic factors. To gain insights into this specialized reproductive development program we used short- and long-read RNA-sequencing (RNA-seq) to study the temporal dynamics of transcript abundance in immuno-cytologically staged barley (*Hordeum vulgare*) anthers and meiocytes. We show that the most significant transcriptional changes in anthers occur at the transition from pre-meiosis to leptotene–zygotene, which is followed by increasingly stable transcript abundance throughout prophase I into metaphase I–tetrad. Our analysis reveals that the pre-meiotic anthers are enriched in long non-coding RNAs (lncRNAs) and that entry to meiosis is characterized by their robust and significant down regulation. Intriguingly, only 24% of a collection of putative meiotic gene orthologs showed differential transcript abundance in at least one stage or tissue comparison. Argonautes, E3 ubiquitin ligases, and lys48 specific de-ubiquitinating enzymes were enriched in prophase I meiocyte samples. These developmental, time-resolved transcriptomes demonstrate remarkable stability in transcript abundance in meiocytes throughout prophase I after the initial and substantial reprogramming at meiosis entry and the complexity of the regulatory networks involved in early meiotic processes.

## Introduction

Due to factors such as global population growth and climate change, food security is a major challenge which can be partly addressed by breeding new crop traits. Breeding relies almost entirely upon meiosis, a specialized form of cell division which generates haploid gametes from diploid parental meiocytes. During the first of two cell divisions in meiosis, parental homologous chromosomes condense, pair, synapse, and undergo recombination within a meiosis-specific protein complex called the synaptonemal complex (SC) (Osman et al., 2011; Naranjo, 2012; Zickler and Kleckner, 2015). The meiotic recombination machinery creates and segregates new allelic combinations which may produce novel traits in offspring. In the life cycle of flowering plants, this pivotal event occurs within specialized reproductive male (stamens) and female (carpel) organs. These reproductive organs are almost entirely composed of vegetative tissues embedding a small number of archesporial cells that differentiate to form meiocytes (Goldberg et al., 1993; Hord and Ma, 2007; Kelliher and Walbot, 2011). Understanding the development of these organs and the biology underpinning meiotic recombination could help to improve the efficiency of trait mapping; potentially leading to improvements in crop breeding.

Phylogenomic, mutagenic, and immuno-cytological tools have been extensively used to clone and characterize plant orthologs of meiotic genes (Lambing et al., 2017). Despite these advances, we still know little about the regulatory mechanisms of gene expression during meiosis. The transcriptomic profile of anthers before, during, and after meiosis has been extensively profiled in *Arabidopsis* (Honys and Twell, 2004; Chen et al., 2010; Yang et al., 2011; Dukowic-Schulze et al., 2014a), rice (Hobo et al., 2008; Fujita et al., 2010; Tang et al., 2010) and maize (Dukowic-Schulze et al., 2014b; Kelliher and Walbot, 2014; Zhang et al., 2014; Yuan et al., 2018; Nelms and Walbot, 2019). These studies provide substantial insights into meiotic transcriptome dynamics in these species with some observations in common. Several of these studies report that many meiotic genes, including ASY1 and DMC1, are both translated and transcribed in pre-meiotic cells—as early as in early archesporial cells in maize (Tang et al., 2010; Kelliher and Walbot, 2014; Zhang et al., 2014; Yuan et al., 2018). This appears to be a highly conserved phenomenon as accumulation of meiotic genes in pre-meiotic cells has also been shown using laser-capture and microdissection of fixed germ cells from human testis (Jan et al., 2017). Early accumulation of meiotic genes appears to be coupled with robust transcriptional changes which may occur at the mitosis-meiosis transition (Yuan et al., 2018), or in a two-step manner during leptotene and early zygotene in maize (Nelms and Walbot, 2019). Female meiocytes are harder to access and isolate in large numbers resulting in fewer studies of gene expression during female meiosis. Instead, Kubo et al. (2013) isolated entire rice ovules at different developmental stages from pre-meiosis to embryo sac and determined their gene expression profiles. This also revealed a major change of the transcriptome in the rice ovule, although later than in the anthers, occurring between zygotene/pachytene and later meiotic stages (Kubo et al., 2013). Other common observations of anther transcriptome dynamics are the significant enrichment of E3 ubiquitin ligases (Fujita et al., 2010; Tang et al., 2010; Yang et al., 2011; Yuan et al., 2018), and mitochondrial processes (Dukowic-Schulze et al., 2014a; Yuan et al., 2018; Nelms and Walbot, 2019) in developing germinal cells.

Although extensively conserved, the meiotic program and gene expression throughout meiosis vary between plant species. Dukowic-Schulze et al. (2014a) compared early meiotic transcripts in *Arabidopsis* and maize anthers finding low correlation in the expression profile of orthologous genes and a number of transcription factors whose expression was unique to *Arabidopsis* or to maize. However, higher correlation in expression profiles was observed when looking at well-defined meiotic genes (Dukowic-Schulze et al., 2014a). Recently, Bélanger et al. (2020) examined the transcriptome of anthers before, during, and after meiosis in both barley and wheat. This uncovered apparent divergence in pre-meiotic expression of a group of 24nt phased small interfering RNAs (phasiRNAs), which are expressed in barley and wheat anthers but not in rice and maize (Bélanger et al., 2020). The anther transcriptome has also been probed by RNA-seq in *Brassica* (Braynen et al., 2017) and sunflower (Flórez-Zapata et al., 2014, 2016) showing a high number of differentially expressed genes when meiocytes were compared to somatic tissue of the same plant or between the cultivated varieties and their wild relative. Meiotic DSB-associated genes were down-regulated in a synthetic autotetraploid *Brassica* compared to its diploid progenitor resulting in abnormal chromosomal segregation (Braynen et al., 2017). On the other hand, compared to its wild relative, meiotic recombination was shown to be higher in cultivated sunflower and many long non-coding RNAs (lncRNAs) were exclusively up-regulated in its meiocytes (Flórez-Zapata et al., 2016). Similarly, low fertility in autotetraploid rice was attributed to differential expression of lncRNAs during anther and ovule meiosis (Li et al., 2020). LncRNAs are poorly characterized compared to protein-coding genes but these studies highlight their potential importance during development (Ponting et al., 2009; Ariel et al., 2015; Shafiq et al., 2016) including their regulatory role during sexual reproduction (Golicz et al., 2018). The processing of a subset of lncRNAs has been shown to generate small RNAs in animals (Jalali et al., 2012) and plants (Ma et al., 2014). Although the regulation of gene expression by small RNA has been reported in cotton anthers (Chen et al., 2018), *Arabidopsis* meiocytes (Huang et al., 2019) and rice ovules (Li et al., 2020), the full integration of small RNAs and lncRNAs biogenesis and function remains to be determined.

Meiocytes are represented by a small number of cells (Kelliher and Walbot, 2011; Zhang et al., 2014) embedded within complex tissues of anthers and ovules making their isolation difficult and time consuming. Several groups have isolated these rare cells and studied their transcriptome. However, most of these studies were restricted to pre-meiosis to zygotene, excluding later meiotic stages (Tang et al., 2010; Dukowic-Schulze et al., 2014a; Kelliher and Walbot, 2014; Zhang et al., 2014; Yuan et al., 2018; Nelms and Walbot, 2019). Unlike the studies where male pre-meiotic cells were recovered by means of microdissection (Tang et al., 2010; Kelliher and Walbot, 2014; Zhang et al., 2014) or protoplasts (Nelms and Walbot, 2019), generally careful staging was not performed for prophase I samples resulting in pooled meiotic stages instead (Chen et al., 2010; Yang et al., 2011; Flórez-Zapata et al., 2014, 2016). Although barley and wheat whole anther transcriptomic profiling has been recently carried out, this did not include specific profiling of expression in meiocytes (Bélanger et al., 2020).

To gain further insight into transcriptional and post-transcriptional regulation in barley reproductive tissues we combined precise immuno-cytological staging with RNA sequencing to analyze transcript profiles of barley anther and meiocyte tissues before, during, and after prophase I. Our data reveal that in barley, as in other plant species, there is extensive transcriptional reorganization between pre-meiosis and early prophase I in anthers. In barley, this switch appears to consist predominantly of down-regulation of many lncRNAs. Sizeable down-regulation of lncRNAs was also observed in the comparison of meiocytes to whole anther tissues in early prophase I. This adds further context to the observed enrichment of lncRNAs in sunflower meiocytes compared to somatic tissues (Flórez-Zapata et al., 2016). As prophase I progressed into metaphase I–tetrad, significant changes to transcript abundance were substantially reduced at each successive stage. In addition, meiocytes showed remarkably stable transcript abundance between leptotene–zygotene and pachytene–diplotene. Further, differential and co-expression analyses indicate the enrichment of several regulatory pathway components in meiocytes in prophase I. These included pentatricopeptide repeat (PPR) RNA binding proteins, E3 ubiquitin ligases, lys48 and NEDD8 specific deubiquitinating enzymes, and small RNA processing components, matching previous observations of enrichment of mitochondrial activity and the ubiquitin proteasome system during meiosis in rice and maize (Tang et al., 2010; Yuan et al., 2018). The details captured in our transcript abundance datasets provide significant insights into this highly organized, stepwise biological process and will lead to both greater understanding and possible applied outcomes.

## Materials and Methods

### Plant Material

Barley cv. Golden Promise plants were grown in cereal compost in a controlled growth room at 18°C for 16h light and 14°C for 8 h dark and 70% humidity. When plants reached the desired stage (pre-meiosis/meiosis) at 5–7 weeks post-germination, they were processed individually by collecting their spikes at the last minute before anther sampling to minimize stress effects.

Male meiotic prophase I in barley progresses over an approximate 36 h time period inside the anthers of developing spikelets (Higgins et al., 2012; Barakate et al., 2014), that are themselves buried within the extending barley culm. To collect appropriately staged meiotic inflorescences, or spikes, we first developed a reliable prediction system based on external morphological characteristics of the main tiller as described for the cultivar Optic (Gómez and Wilson, 2012). In our environmentally conditioned growth room, the barley cultivar Golden Promise plants reached meiosis at 6 weeks post-germination, when the flag leaf was emerging, and the spikes were 0.5–2 cm in length. As we had previously determined a strong correlation between anther length and meiotic progression (Arrieta et al., 2020), anthers were dissected under a stereomicroscope and measured before being stored as length classes in RNA*later*^TM^ (Thermo Fisher Scientific). For each length class, a subset of 10 anthers was stored in PBS buffer and used for chromosomal acetocarmine staining for meiotic staging. This allowed us to determine the precise correlation between anther size and meiotic stage in this material.

Surfaces and tools were cleaned with 70% Ethanol and RNase AWAY^TM^ (Thermo Fisher Scientific) prior to material collection and all steps were done wearing gloves. The collected spikes were placed in a Petri dish with wet filter paper (using RNase-free milli-Q water) on a cool pack or ice during the sampling, including under the stereomicroscope with LED light to avoid RNA degradation. Fine tweezers were used to remove awns and collect anthers of all florets between the fifth and fifteenth position from the bottom under a stereomicroscope resulting in 60 anthers on average, a process that takes about 10 min. All materials were collected in the afternoon between 1:00 p.m. and 3:00 p.m. only to improve sample homogeneity. Four stages were collected: pre-meiosis–G2, leptotene–zygotene, pachytene–diplotene and all stages from metaphase I to tetrad.

Each barley floret contains three synchronized anthers. One anther was staged using acetocarmine staining and the two others were collected in 1.5 ml Eppendorf tube containing 100 μl RNA*later*^TM^. Anthers of the same length and meiotic stage were pooled together. From this tube, 10 random anthers were transferred into a new tube with 1x PBS buffer for staging validation with immunostaining using meiosis specific antibodies (Supplementary Method 1). The process was repeated until at least three replicates of 60–200 anthers were collected for each meiotic stage.

**FIGURE 1:**
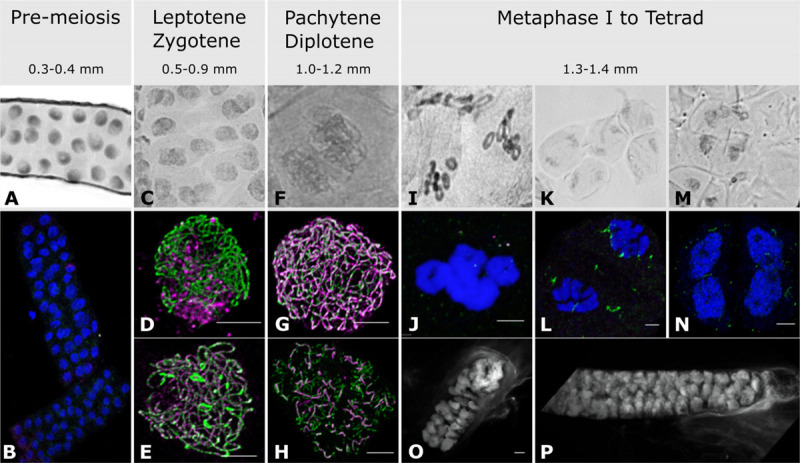
Anther and meiocyte collection and staging. Anthers were collected according to their size and staged with both acetocarmine **(A,C,F,I,K,M)** and immuno-cytology **(B,D,E,G,H,J,L,N). (A,B)** Pre-meiotic stage; **(C)** leptotene/zygotene stage; **(D)** leptotene stage; **(E)** zygotene stage; **(F)** pachytene stage; **(G)** pachytene stage; **(H)** diplotene stage; **(I,J)** metaphase I; **(K,L)** anaphase I; **(M,N)** tetrads; **(O,P)** isolated fresh meiocyte clusters at leptotene/zygotene and pachytene/diplotene, respectively. Acetocarmine (Gray), DAPI (Blue and White for images **O,P**), ASY1 (Green), ZYP1 (Magenta). Scale bar 5 μm. Note images **(O,P)** have associated videos.

To prepare meiocyte samples, 30–50 anthers that were staged as leptotene–zygotene and pachytene–diplotene by immuno-cytology were used to release meiocyte clusters onto microscope slides (Supplementary Method 2). A sample of these were stained with DAPI to evaluate the purity of the preparation (Figures 1O,P and Supplementary Videos 1, 2) and the remaining material was transferred into Eppendorf tubes containing TRIzol^®^ for subsequent total RNA isolation. Similar to anther samples, meiocyte clusters at close stages of leptotene and zygotene or pachytene and diplotene were mixed in equal proportions. The duration of entire meiocyte isolation process for each replicate depends on anther stage and took on average 1.5 h.

For comparison we included four samples of germinating embryo (EMB) as those have been shown to represent a wide range of transcripts [International Barley Genome Sequencing Consortium (IBGSC), 2012]. Barley cultivar (cv.) Golden Promise seeds were germinated in a Petri dish on wet paper for 5 days in dark. Three EMB were collected per sample by removing the residual seed material. In total four samples were collected and used for total RNA extraction.

### Cytology and Imaging

Ten anthers of each pool were fixed in 4% formaldehyde (1x PBS/0.5% Triton^TM^ X-100/0.5% Tween 20) for 20 min, rinsed twice in 1x PBS and ground gently in the tube with a disposable pestle. Tubes were centrifuged at 4,500 rpm for 1 min and 20 μl meiocytes suspension was transferred onto a *Polysine*^TM^ 2 (*Poly-L-Lysine coated slides*) and left to air dry. The immuno-cytology (Supplementary Method 1) was done according to Colas et al. (2017).

### Barley Anther Transcriptome (BAnTr) Construction

Total RNA was extracted from the 12 anther samples, six meiocyte samples and 4 EMB and sequenced using both Illumina NextSeq 550 and PacBio Sequel v1 platforms (Supplementary Method 3).

#### Illumina Read Mapping

The resulting Illumina sequencing reads were mapped individually against the barley *cv.* Golden Promise assembly using STAR 2.7.1a (Dobin et al., 2013) in a two-step process based on Veeneman et al. (2016). The first genome index was generated using default settings. The exact parameters for the two-step mapping process can be found in Supplementary Method 4.

#### PacBio Read Mapping

All three samples of long-read transcriptome data were processed individually using the SMRT Analysis software package v3.1.0^1^ for building of the consensus circular reads (CCS) and demultiplexing which included barcode and primer removal. Afterward all transcripts were merged for the clustering and polishing. Polishing on the merged transcripts was done using proovread v.2.14.1 (Hackl et al., 2014) with the Illumina reads from the 12 anther samples and the 6 meiocyte samples. The polished transcripts were mapped to the Golden Promise reference assembly using GMAP version 2018-07-04 (Watanabe and Wu, 2005).

#### Transcriptome Building

We used the Mikado pipeline (Mikado v1.2.4; Venturini et al., 2018) to join the different strands of transcriptome evidence, generated above. As additional transcript evidence from a more complete transcriptome we included the BaRTv1 transcripts (Rapazote-Flores et al., 2019). The BaRTv1 transcripts were mapped to the Golden Promise reference assembly (GMAP version 2018-07-04; -n 0 –min-trimmed-coverage = 0.80 –min-identity = 0.90). Input files for the Mikado file included a splice junction file generated by Portcullis (Mapleson et al., 2018; default parameters), open reading frame identification of the transcripts by TransDecoder^2^ (default parameters; Haas et al., 2013) and blast results using DIAMOND BLASTx (Buchfink et al., 2015; –evalue 1e-5) against the NCBI non-redundant protein database as evidence. Together with the stringtie.gtf, scallop.gtf, pacbio.gtf and bartv1.gtf those were combined and scored, generating the Barley Anther Transcriptome (BAnTr). This dataset comprises 65,795 genes and 119,778 transcripts (Supplementary File 1: AntherTranscriptomeBAnTr.fasta at http://doi.org/10.6084/m9.figshare.12136773).

A padded version of the transcriptome was generated for the transcript quantification as previous experiments in *Arabidopsis* showed this to improve quantification and provide more similar results to high-resolution real-time PCRs (Zhang R. et al., 2017; Supplementary File 2: AntherTranscriptomeBAnTrPadded.fasta at http://doi.org/10.6084/m9.figshare.12136773).

To distinguish between protein-coding and long non-coding transcripts, all transcripts were run through the NCBI ORF finder. Transcripts and corresponding genes were assigned as coding, if one hit was identified in a DIAMOND BLASTp (v0.9.24; Buchfink et al., 2015) search [–query-cover 60 –evalue 1e-30] against the NCBI non-redundant protein database (Supplementary File 3: AntherProteomeBAnTr.fasta at http://doi.org/10.6084/m9.figshare.12136773). Additionally, transcripts were checked using CPC2 (coding potential calculator 2, run on both strands) and InterProScan (v5.39; default parameters; Jones et al., 2014). If both supported a coding sequence, those were also included in the coding transcripts; if only one of those provided evidence for coding, the sequences were allocated to the unclassified group. All remaining transcripts and their corresponding genes were assigned as long non-coding RNA. For further classification we used the approach from Zhang Z. et al. (2020) by comparing the location of lncRNA to the location of protein-coding RNA using Cuffcompare (Trapnell et al., 2012). In addition, we also compared the lncRNA location with the transposable element location. Based on this lncRNA were then classified into TE-lncRNA, intergenic lncRNA (cuffcompare class code u), intronic lncRNA (cuffcompare class code i), sense lncRNA (cuffcompare class code o), antisense lncRNA (cuffcompare class code x) and other lncRNA (remaining cuffcompare class codes). Conservation of BAnTr lncRNA transcripts was assessed by alignment using BLAST (Altschul et al., 1990) to: RiceLncPedia transcripts (Zhang Z. et al., 2020); *Arabidopsis* non-code v5 transcripts (Zhao et al., 2016); maize, wheat, and *Brachypodium* GreeNC transcripts (Paytuví Gallart et al., 2016); and barley CANTATAdb v2.0 transcripts (Szcześniak et al., 2019).

### Differential Expression

To study differential gene expression the 12 anther and 6 meiocyte Illumina sequencing samples were mapped against our above described padded BAnTr transcriptome using Salmon v.0.14.1 (Patro et al., 2017; -validateMappings –useVBOpt –seqBias –gcBias –posBias). The subsequent analysis was conducted using the 3D RNA-seq pipeline (Guo et al., 2019; accessed 28/11/2019). This pipeline is a combination of different R packages. Transcript abundance is imported using the lengthScaledTPM method from tximport (Soneson et al., 2016) and transformed into gene level counts or kept as transcript level counts. Low expressed transcripts with a count per million reads (CPM) below 1 in fewer than three samples were removed. This reduced the dataset to 31,918 genes and 50,861 transcripts. Gene and transcript read counts were normalized using the trimmed mean of M values (TMM) method, implemented in the edgeR package (Robinson et al., 2010; Robinson and Oshlack, 2010; McCarthy et al., 2012). Based on the experimental design, six contrast groups were built: anthers at leptotene–zygotene versus anthers at pre-meiosis; anthers at pachytene–diplotene versus anthers at leptotene–zygotene; anthers at metaphase I–tetrad versus anthers at pachytene–diplotene; meiocytes at leptotene–zygotene versus anthers at leptotene–zygotene; meiocytes at pachytene–diplotene versus anthers at pachytene–diplotene; and meiocytes at pachytene–diplotene versus meiocytes at leptotene–zygotene. Differential expression analysis was done using the voom function in the limma R package (Law et al., 2014; Ritchie et al., 2015). Genes and transcripts were rated as differentially expressed with a log_2_-fold change (log_2_FC) above 1 or below −1 and an adjusted *p*-value (by the Benjamini-Hochberg method; Benjamini and Hochberg, 1995) of below 0.01. To analyse the relationship between replicates, a multidimensional scaling plot was generated using the transcript expression data.

#### Differential Alternative Splicing Analysis

Differential alternative splicing (DAS) analysis is part of the 3D RNA-seq pipeline. It integrates the diffSplice function from the limma R package and compares the log_2_FC of each individual transcript with the gene level log_2_FC. Transcripts with a Δ percent spliced (ΔPS) ratio of above 0.1 were assigned as significant alternative spliced transcripts. To determine the alternative splicing events, we used SUPPA version 2.3 (Alamancos et al., 2015; Trincado et al., 2018).

#### Co-expression Analysis

To identify co-expressed networks, we used the weighted gene co-expression network analysis (WGCNA) R package (v1.63; Langfelder and Horvath, 2008). The same reduced dataset of 31,918 expressed genes as introduced above was used as input. For the network construction an approximate scale-free topology of above 0.85 was achieved with a soft power of 10. Network construction and module assignment was done with the following settings: a signed hybrid network, mergeCutHeight of 0.35, a minModuleSize of 30 and a deepSplit of 4.

#### Gene Ontology and Functional Annotation

Multiple strands of evidence were combined for the functional annotation of the proteins. Mercator4 v.2 (Schwacke et al., 2019) was used to classify the proteins into functional bins. eggNOG (Huerta-Cepas et al., 2017; Huerta-Cepas et al., 2019) was used to add COG (Clusters of Orthologous Groups of proteins) annotation and a protein description. PANNZER2 (Törönen et al., 2018) was used for the GO term identification and a protein description. The GO enrichment analysis was done using the topGO R package using the fisher weight01 algorithm and a *P*-value cut-off below 0.001 (Alexa et al., 2006). TopGO only outputs an exact *P*-value until 1e-30. Everything below will be given as *P* < 1e-30. For visual presentation in the GOplot R package (Walter et al., 2015) those were set to 1e-30.

## Results

### Accurate Staging of Anthers

As meiosis is not completely synchronized along the length of a spike (Lindgren et al., 1969), only anthers from the middle spikelets were collected. In total, three biological replicates with at least 120 anthers in each were collected for pre-meiosis (0.3–0.4 mm anthers), leptotene–zygotene (0.5–0.9 mm anthers), pachytene–diplotene (1.0–1.2 mm anthers) and metaphase I–tetrad (1.3–1.4 mm anthers) (Figure 1).

Acetocarmine stained pre-meiotic spreads showed small nuclei (Figure 1A) without any labeling by antibodies against the SC proteins HvZYP1 and TaASY1, although a signal can be detected with anti-TaASY1 mainly in the cytoplasm (Figure 1B). Contrary to the pre-meiotic stages, anther size and acetocarmine staining were not entirely reliable for leptotene–zygotene staging (Figure 1C) but immunostaining with antibodies against TaASY1 and HvZYP1 allowed us to easily distinguish between these two close developmental stages. At leptotene, linear ASY1 axes are formed while there is no or little labeling of HvZYP1 at the initiation of synapsis (Figure 1D). Zygotene on the other hand was determined by the presence of HvZYP1 labeling during synapsis progression as previously described (Colas et al., 2017; Figure 1E). Pachytene and diplotene were somewhat easier to find based on anther size and acetocarmine spreads (Figure 1F) and were distinguished using antibodies against TaASY1 and HvZYP1 proteins (Figures 1G,H). Pachytene (Figure 1G) was characterized by the polymerization of HvZYP1 between the ASY1 axes along the entire chromosomes and diplotene (Figure 1H) was identifiable by the appearance of tinsel-like chromosomal structures as previously described (Colas et al., 2017). However, this immuno-cytological work revealed that meiocytes within the same anther were not fully synchronized and we opted for combining developmentally close samples to make mixtures of equal proportions of leptotene and zygotene or pachytene and diplotene. Later stages from metaphase I to tetrads were combined into a single sample (Figure 1). Metaphase I is characterized by the presence of seven ring bivalents (Figures 1I,J) while anaphase I (Figures 1K,L) and anaphase II samples show chromosomes or chromatin segregation, respectively (Colas et al., 2017). Anther samples of these later stages were not as homogenous as those at prophase I (leptotene–diplotene), containing both metaphase I and anaphase I or both anaphase II and telophase II. Very few anthers contained tetrads (Figures 1M,N) and none reached pollen stage.

To prepare meiocyte samples, a subset of anther samples that were staged as leptotene–zygotene and pachytene–diplotene by immuno-cytology were used to release meiocyte clusters onto microscope slides. A sample of these were stained with DAPI to evaluate the purity of the preparation (Figures 1O,P and Supplementary Videos 1, 2) and the remaining material was transferred into Eppendorf tubes containing TRIzol^TM^ for subsequent total RNA isolation. The RNA integrity numbers (RIN) were in the range of 5.8–7.3 and 7.3–9.7 for meiocyte and anther samples, respectively. Similar to anther samples, meiocyte clusters at close stages of leptotene and zygotene or pachytene and diplotene were mixed in equal proportions.

The combination of anther size and immuno-cytology allowed us to delineate different developmental stages. We extracted total RNA and sequenced 18 samples (3 biological replicates of 2 meiocyte and 4 anther stages) generating a total of 2.1 billion reads (Supplementary Table 1). For comparative purposes we extracted and sequenced RNA from 4 replicate germinating embryo (EMB) samples generating over 245 million reads.

Creation of a reproduction-inclusive barley transcriptome reference assembly For RNA-seq and differential gene expression analysis we first developed a reference Barley Anther Transcriptome (BAnTr). We did this for two important reasons. First, we anticipated that anther/meiocyte tissues potentially contain a set of very specific transcripts. So, to complement the Illumina short read RNA-seq data, we generated and included three 5′ cap- and 3′ polyA-captured PacBio IsoSeq datasets from a mixture of the RNA samples mentioned above. Second, we are working with the transformation reference barley cultivar Golden Promise and had recently generated a Golden Promise genome reference assembly (Schreiber et al., 2020). Building a reference-guided transcriptome based on the same cultivar will be more complete and more accurate as cultivar specific transcripts will be included. To include as many barley transcripts as possible, the Illumina RNA-seq and IsoSeq reads of anthers and meiocytes were combined with the short reads from the germinated embryo samples and ultimately with the BaRTv1-Reference Transcript Dataset reported recently by Rapazote-Flores et al. (2019). The final transcriptome, BAnTr, comprised 65,795 genes and 119,778 transcripts.

For each sample, transcript quantification files were generated using Salmon (Patro et al., 2017) in conjunction with BAnTr. The output quantification files were then read into the 3D RNA-seq pipeline (Guo et al., 2019) to generate read counts which were converted to transcript per million (TPM) using the tximport R package (Soneson et al., 2016). The data used as input for the 3D RNA-seq pipeline has been deposited at figshare: https://doi.org/10.6084/m9.figshare.11974182. Read counts and TPMs were pre-processed and filtered to reduce noise and technical variance by excluding low abundance transcripts (keeping transcripts with a count per million reads ≥ 1 in at least 3 samples, and genes where at least one transcript passed the filtering). After filtering, 50,861 transcripts encoded by 31,918 genes remained. A multidimensional scaling plot was generated showing clear separation between samples (Supplementary Figure 1). Using differentially expressed genes (DEGs) criteria described in section “Materials and Methods,” a total of 10,713 DEGs (Supplementary Figures 2A,B) were identified and for further analysis divided into three categories based on protein-coding potential: protein-coding, long non-coding RNA (lncRNA) (Supplementary Figure 2C), and unclassified (see section “Materials and Methods”). phasiRNAs were detected in isolated meiocytes and anthers of maize (Zhai et al., 2015; Dukowic-Schulze et al., 2016). Using several male sterile mutants defective in specific anther cell layers, Zhai et al. (2015) revealed the spatiotemporal dynamics of 21 and 24 nt phasiRNAs highly accumulated at pre-meiotic and meiotic stages, respectively. PhasiRNAs have been shown to function in DNA methylation during anther development playing a critical role in fertility (Liu C. et al., 2020; Zhang M. et al., 2020). More recently, Teng et al. (2020) demonstrated that *Dicer-like 5* generates the 24 nt phasiRNAs in meiotic anthers of maize. Production of phasiRNAs is triggered by cleavage of non-coding precursor (PHAS) transcripts directed by micro RNAs (miRNAs) miR2118 and miR2275, to produce 21 and 24 nt phasiRNAs, respectively (Johnson et al., 2009; Song et al., 2012a). To identify putative PHAS transcripts, all lncRNA transcripts were assessed for miR2118 and miR2275 recognition sites using psRNATarget (Dai et al., 2018). This identified 568 lncRNAs with predicted miR2118 recognition sites, 553 lncRNAs with predicted miR2275 recognition sites, and 53 predicted to contain recognition sites for both miRNAs. Matching the overall pattern of lncRNA expression, putative targets of both miR2118 and mi2275 were predominantly expressed in anthers and not in germinating embryos (93 and 86%, respectively). Expression of putative miR2275 targets (Supplementary Figure 2E) was more stable than expression of putative miR2118 targets (Supplementary Figure 2D) with 74 and 46% of each showing differential expression in any comparison, respectively. However, putative targets of these miRNAs which were differentially expressed displayed a broadly similar pattern of expression across sample stages (Supplementary Figures 2F,G), predominantly characterized by down-regulation from pre-meiosis to pachytene–diplotene. A small proportion—1% of each—of these lncRNAs were enriched in meiocytes compared to whole anthers.

Several analyses were used to further probe the functional pathway and developmental expression of this high number of DEGs. However, in our analysis we focused on meiotic recombination and gene regulation pathways.

### Co-expression Analysis Detected Four Modules Enriched in Meiocyte Samples

**FIGURE 2:**
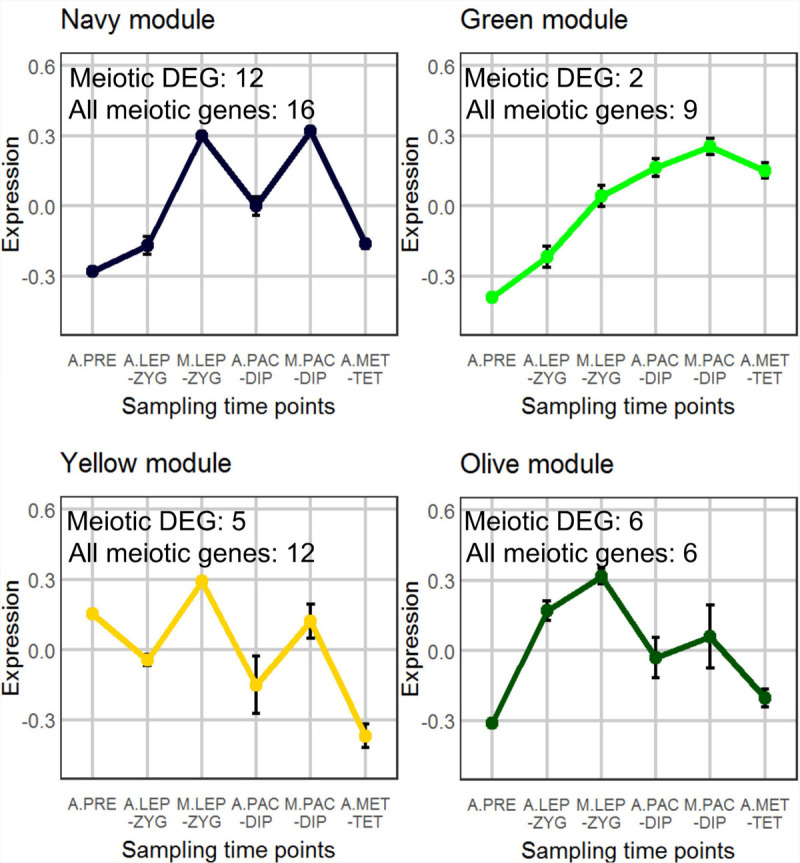
WGCNA analysis of co-expressed genes. A total of 17 modules were found and the selected four show an interesting pattern for meiocyte enriched genes. The samples (3 replicates each) are A.PRE, anther pre-meiosis; A.LEP-ZYG, anther leptotene–zygotene; M.LEP-ZYG, meiocyte leptotene–zygotene; A.PAC-DIP, anther pachytene–diplotene; M.PAC-DIP, meiocyte pachytene–diplotene; A.MET-TET, anther metaphase I–tetrad. The prefixes A. and M. in the sample names depict anther and meiocyte samples, respectively.

**FIGURE 3:**
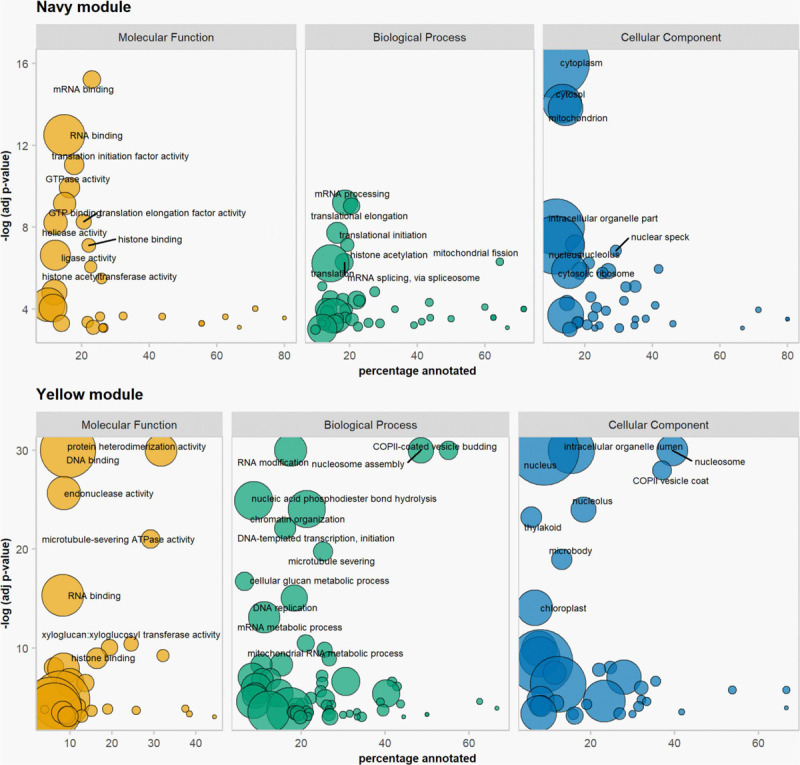
Gene ontology enrichment analysis. Results from the navy and the yellow modules of the WGCNA. Size of bubbles correspond to total number of proteins associated with the GO term.

We used the weighted gene co-expression network analysis (WGCNA) tool (Langfelder and Horvath, 2008) to identify co-expression networks within all 31,918 expressed genes in the anther and meiocyte dataset. The analysis identified 17 gene clusters (shown as color-coded modules in Supplementary Figure 3) with an additional un-clustered gray module (Supplementary Table 2). The number of genes in each module ranged from 38 to 6209 (Supplementary Table 2). Variation was also evident in differential eigengene network analysis showing levels of correlation between clusters (Supplementary Figure 4). WGCNA navy and yellow modules were enriched in the meiocyte samples in comparison to anthers. In total, four modules (navy, yellow, green, and olive) showed higher expression in isolated meiocytes in comparison to anthers (Figure 2). Gene ontology (GO) enrichment analysis highlighted mRNA and RNA binding GO terms as significantly enriched in the navy module (*P* = 6E-16 and *P* = 3.1E-13); the yellow module was significantly enriched for DNA binding (*P* < 1E-30), RNA modification, and nucleosome GO terms (Figure 3 and Supplementary Table 3) (*P* < 1E-30 and *P* < 1E-30). From the remaining modules, genes clustered in the red module showed a pattern of expression which was approximately inverse to genes clustered in the navy module (Supplementary Figure 3). The red module is significantly enriched in genes located in chloroplast components (Supplementary Figure 5 and Supplementary Table 3) (chloroplast thylakoid membrane, *P* = 2.2E-13 and chloroplast envelope, *P* = 3.7E-6). The blue and the light-green modules show a similar downward trend of expression with meiotic progression (Supplementary Figure 3).

### Large Scale Transcriptional Re-programming in Anthers at Meiosis Entry Is Followed by Progressive Reduction of Significant Changes Into Metaphase–Tetrad Stage

**FIGURE 4:**
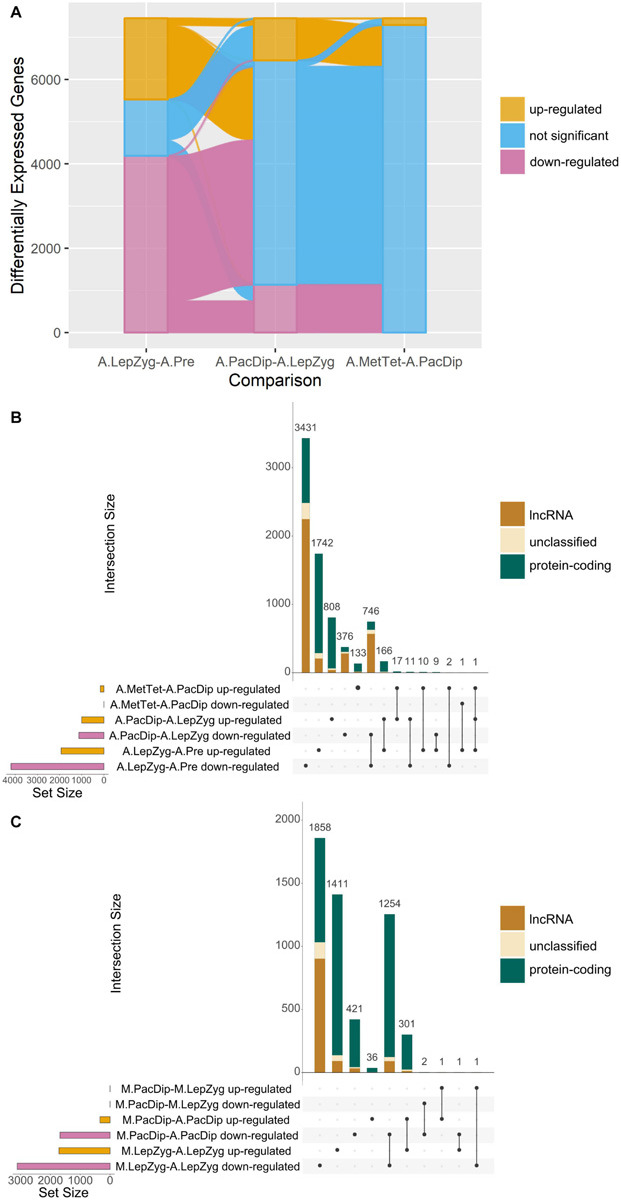
Comparisons of differential gene expression. **(A)** Alluvial plot showing up- or down-regulation of genes significantly differentially expressed in all anther (A) meiotic stage comparisons. The comparisons are: A.LepZyg-A.Pre, leptotene–zygotene versus pre-meiosis; A.PacDip-A.LepZyg, pachytene–diplotene versus leptotene–zygotene; and A.MetTet-A.PacDip, metaphase I–tetrad versus pachytene–diplotene. The bar at each stage represents the total complement of genes which are differentially expressed in any anther comparison, the bar is colored by the proportion of genes in each successive meiotic stage comparison on the x axis which is up-regulated (yellow), down-regulated (pink), or not significant (blue). The lines connecting the bars between x axis comparisons represent movement of genes from one expression category to another between successive meiotic stage comparisons. The vast majority of all DEGs (pink and yellow) at any stage are present in the comparison of leptotene–zygotene anthers to pre-meiotic anthers. A large proportion of DEGs in the first comparison do not show a significant change in expression between pachytene–diplotene and leptotene–zygotene, as represented by the pink and yellow lines connecting to the blue shaded area in the second *x*-axis comparison. **(B)** UpSet plot of the same anther meiotic stage comparisons and **(C)** UpSet plot of meiocyte (M) stage and meiocyte-anther tissue comparisons. UpSet plots are similar in principal to the more commonly used Venn and Euler diagrams in that they represent the intersection of distinct groups (sets) but are better at representing large numbers of groups (Lex et al., 2014). The total number of genes in each group (the set size) is represented by a histogram on the left-hand side of the *x*-axis. The black dots underneath the *x*-axis indicate which groups are represented in the histogram above showing the number of genes represented by this group. Where only one dot is present the histogram above represents the number of genes unique to this group. Where two or more dots are connected by a line the histogram above represents the number of genes which occur in the connected groups. The comparisons are: M.PacDip-M.LepZyg, meiocytes at pachytene–diplotene versus meiocytes at leptotene–zygotene; M.LepZyg-A.LepZyg, meiocytes at leptotene–zygotene versus anthers at the same stage; M.PacDip-A.PacDip, meiocytes at pachytene–diplotene versus anthers at the same stage. The prefixes A. and M. in the sample names depict anther and meiocyte samples, respectively.

**FIGURE 5:**
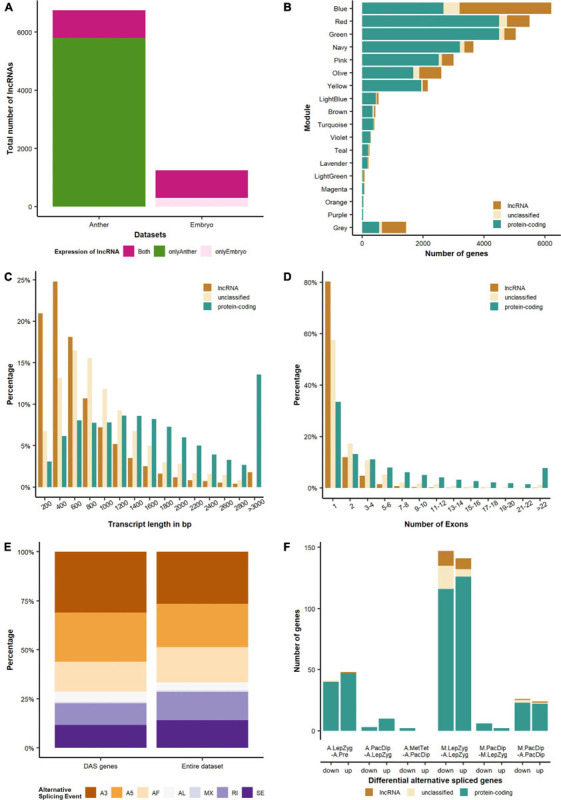
Differential expression and alternative splicing of different gene categories. **(A)** Compared with germinating embryos, anthers are enriched in lncRNAs. **(B)** Distribution of coding, non-coding and undefined genes in different modules. The modules Violet, Turquoise, Navy, Green, Yellow and Olive are enriched in meiocytes. **(C)** Length and **(D)** exon number distributions of different transcript categories. **(E)** Distribution of alternative splicing events for the entire dataset and the DAS (differential alternative spliced) genes. A3, alternative 3′ splice-site; A5, alternative 5′ splice-site; AF, alternative first exon; AL, alternative last exon; MX, mutually exclusive exons; RI, retained intron; SE, skipping exon. **(F)** Differential alternative spliced genes. The comparisons are: A.LepZyg-A.Pre, anther leptotene–zygotene versus anther pre-meiosis; A.PacDip-A.LepZyg, anther pachytene–diplotene versus anther leptotene–zygotene; A.MetTet-A.PacDip, anther metaphase I–tetrad versus anther pachytene–diplotene; M.LepZyg-A.LepZyg, meiocyte leptotene–zygotene versus anther leptotene–zygotene; M.LepZyg-M.LepZyg, meiocyte leptotene–zygotene versus meiocyte leptotene–zygotene; M.PacDip-A.PacDip, meiocyte pachytene–diplotene versus anther pachytene–diplotene. The prefixes A. and M. in the sample names depict anther and meiocyte samples, respectively.

3D RNA-seq analysis revealed a dramatic alteration in gene expression in anthers between pre-meiotic and leptotene–zygotene stages followed by progressively fewer significant changes in pachytene–diplotene and metaphase–tetrad stages (Figures 4A–C and Supplementary Table 4). The number of DEGs was highest (*n* = 6,119) in comparison of anthers at the leptotene–zygotene stage versus pre-meiosis. Strikingly, 48% of the total number of DEGs and 65% of the down-regulated DEGs are lncRNAs in this comparison (Figures 4A,B). Only 8.6% (*n* = 2,024) of these lncRNA transcripts returned an alignment with rice, maize, *Arabidopsis*, wheat, or *Brachypodium* lncRNA database sequences. When BAnTr lncRNA transcripts were aligned to barley CANTATAdb lncRNA data transcripts the number returning alignment increased to 30.45%. No clear relationship between lncRNA classification and successful alignment was observed (Supplementary Table 5). Compared with germinating embryos, anthers contain a much larger number of lncRNAs represented mainly in bleu module (Figures 5A,B). As expected, the majority of these lncRNAs are relatively short (Figure 5C) and comprise a single exon (Figure 5D). In anther tissues, there was a further pronounced, but much smaller, change in gene expression at pachytene–diplotene versus leptotene–zygotene (Figures 4A,B). However, gene expression in meiocytes appears stable between leptotene–zygotene and pachytene–diplotene stages, with only 4 DEGs (2 up-regulated; 2 down-regulated) observed in this comparison (Figure 4C). Only 164 genes (163 up-regulated; 1 down-regulated) are significantly differentially expressed in anthers at the metaphase I–tetrad stage compared to the pachytene–diplotene stage (Figures 4A,B). When lncRNAs are excluded from the analysis, the largest number of DEGs occurs in meiocytes at the leptotene–zygotene stage when compared to anthers at the same stage (*n* = 3,517; Figure 4C); the number of protein-coding DEGs in comparison of anthers at the leptotene–zygotene stage versus pre-meiosis is 2710 (Figure 4B).

### Differential Alternative Splicing

A striking feature of many of the genes that are putatively involved in meiosis and recombination in barley is the number of introns and exons they contain. The average of our protein-coding transcripts in the BAnTr transcriptome is 7.5 exons per gene. Based on the literature and phylogenetic analysis, we compiled a list of 121 barley genes with confirmed role in meiosis in yeast, animal and plant species. Our selection of 121 potential meiotic genes has an average of 25 exons per gene—more than three times the average for all genes. One possible reason may be that regulation of these genes occurs at the post-transcriptional level through delayed pre-mRNA processing or DAS. Indeed, 84 out of the 121 potential meiotic genes have more than one isoform. However, only two of those genes are significantly differentially alternatively spliced (HvHSP90.7 and HvPTB1a). HvHSP90.7 has two main expressed isoforms throughout meiosis, BAnTr.GP.7HG019194.1 and BAnTr.GP.7HG019194.16 with the first showing no significant expression changes and the second being significantly upregulated in the step from pre-meiosis to leptotene-zygotene. BAnTr.GP.7HG019194.16 is characterized by an alternative 3′ splice site of 11 nucleotides which causes a premature stop codon. HvPTB1a has multiple isoforms expressed throughout meiosis, but only two transcripts (BAnTr.GP.4HG005942.17 and BAnTr.GP.4HG005942.18) are differentially expressed and one BAnTr.GP.4HG005942.1 shows differential transcript usage. BAnTr.GP.4HG005942.18 is characterized by an additional exon and BAnTr.GP.4HG005942.17 retains an intron. Both cases result in premature stop codons.

The analysis of DAS genes across the whole transcriptome revealed that most contain isoforms with different 3′ or 5′ splice-sites, representing 48% of the total identified events (Figure 5E). Only 0.8% of the genes contained isoforms with mutually exclusive exons. The DAS genes were similarly distributed with no splicing event being strongly enriched in comparison to the whole dataset. GO enrichment analysis showed for both groups of DAS genes, i.e., with alternative 3′ or 5′ splice-sites, an enrichment in ribonucleoprotein complex (*P* = 2.8E-4 and *P* = 2.6E-5) which contains the small nuclear ribonucleoprotein particles (snRNPs) forming the spliceosome (Supplementary Table 3). We also observed enrichment of the GO terms U2-type prespliceosome (*P* = 7.3E-5) and U1 snRNP (*P* = 1.3E-4) and could identify both U2A-factor (BAnTr.GP.5HG019656) and U1-70k (BAnTr.GP.1HG004326, BAnTr.GP.1HG006488) components plus additional genes involved in forming the small nuclear ribonucleoprotein particle (snRNP). The most differentially alternatively spliced genes can be observed in the comparison of meiocytes to anthers in leptotene–zygotene stage (Figure 5F). GO enrichment analysis of anther depleted genes highlighted the same GO terms as above (U2-type prespliceosome, *P* = 7E-5 and U1 snRNP, *P* = 1.1E-4). The GO term “regulation of DNA damage checkpoint” (*P* = 2.5E-4) was enriched in meiocytes (Supplementary Table 3). This GO term was assigned to replication Protein A 2A (RPA2A) and two other WD40 repeat containing proteins, one of which was annotated as DNA damage-binding protein 2 (DDB2). Surprisingly, HvRPA2A was not enriched in meiocytes at either stage while DDB2 and the other WD40 repeat containing protein showed significant (*P* = 1.5E-4 and *P* = 1.3 E-3) up-regulation in meiocytes compared to anthers at leptotene, although did not exceed our threshold log fold change value of + 1.5.

### Genes Annotated With Roles in Post-transcriptional and Post-translational Modification Are a Major Component of Genes Enriched in Prophase I Meiocytes

GO enrichment analysis of DEGs points to a sizeable role for RNA and post-translational modifications in prophase I (Supplementary Table 3). This was most acutely highlighted in the comparison of meiocytes to anthers at leptotene–zygotene—representing the largest contrast in expression of protein-coding genes. Lys48-specific deubiquitinase activity (GO:1990380; *P* = 0.00083), histone lysine demethylation (GO:0070076; *P* = 0.00068), histone H3-K9 demethylation (GO:0033169; *P* = 0.00074), ligase activity (GO:0016874; *P* = 0.0002), and RNA modification (GO:0009451; *P* = 3.60E-10) were all significantly enriched in meiocytes. Enrichment of the RNA modification GO term reflected up-regulation of genes annotated as pentatricopeptide repeat (PPR) proteins, which regulate gene expression at the RNA level (Manna, 2015). Up-regulation of these genes also drives significant enrichment of endonuclease activity (GO:0004519; *P* = 0.00088) in this comparison. Of the two genes enriched in meiocytes at pachytene–diplotene versus leptotene–zygotene one was annotated as a PPR protein, the other was unannotated, and both depleted genes in this comparison were lncRNAs. Continued enrichment of these genes at pachytene–diplotene in meiocytes versus anthers at the same stage is also reflected in significant enrichment of mRNA binding (GO:0003729; *P* = 0.00033). Meiocyte enriched genes in this contrast group were enriched for GO terms including helicase activity (GO:0004386; *P* = 0.00088) and synapsis (GO:0007129; *P* = 0.00023), reflecting the formation of the SC in meiocytes at this stage. GO enrichment of ligase activity in DEGs in meiocytes at leptotene–zygotene compared to anthers at the same stage is driven by meiocyte enrichment in E3 ubiquitin ligases. In total, 890 ubiquitin or ubiquitin-like (SUMO, NEDD8) E3 ligases were annotated in the BAnTr transcriptomic dataset; in our dataset 133 of these were specific to anther and meiocyte tissues, 63 were unique to germinating embryos, and 589 were expressed in both anther and germinating embryo tissues. 166 of these were differentially expressed in a least one contrast group and 71 were enriched in meiocytes compared to anthers at the same stage (Supplementary Table 6). Notably, those enriched in meiocytes included many E3 ligases—or multi-subunit E3 ligase components—with confirmed roles in meiosis in other organisms. These included: a SKP1 ortholog (BAnTr.GP.5HG012508) and three F-box proteins (BAnTr.GP.2HG002834, BAnTr.GP.3HG014344, BAnTr.GP.3HG014348) which form part of the multi-subunit SKP Cullin F-box (SCF) E3 ligase complex; one RING-H2 component of the anaphase promoting complex (BAnTr.GP.1HG006974); and three seven-*in absentia* (SINA) E3 ligases (BAnTr.GP.2HG018292, BAnTr.GP.3HG000774, BAnTr.GP.3HG000850). HEI10, a highly conserved E3 ubiquitin/SUMO ligase which is known to be involved in DSB repair (Ziolkowski et al., 2017), was stably expressed in all tissues and stages, including in germinating embryo tissues. Although no NEDD8 E3 ligases are differentially expressed in any stage or tissue comparison, NEDD8-specific protease activity (GO:0019784; *P* = 0.00029) is enriched in pachytene–diplotene compared to leptotene–zygotene in anthers. Significant up-regulation of Lys48 specific deubiquitinase activity, in parallel to E3 ligase activity, might reflect delicate regulation of the ubiquitination cascade at this stage. However, of the four significantly up-regulated protein-coding genes assigned this GO by PANNZER2, only one (BAnTr.GP.7HG015226) is explicitly annotated as a deubiquitinating enzyme (DUB); two are annotated as B-box transcription factors and the other as an O-fucosyltransferase. Lys48 deubiquitination (GO:0071108; *P* = 3.20E-5) is also enriched in pachytene–diplotene compared to leptotene–zygotene in anthers reflecting the up-regulation of four protein-coding genes annotated as otubain-like DUBs, active on Lys48-linked polyubiquitin chains and NEDD8 (Edelmann et al., 2009). Histone lysine demethylation and histone H3-K9 demethylation enrichment in meiocytes compared to anthers at leptotene–zygotene is driven by up-regulated protein-coding genes orthologous to argonaute (AGO) proteins and demethylases KDM3 (BAnTr.GP.1HG003366), PKDM9 (BAnTr.GP.UnG004036), JMJ705 (BAnTr.GP.UnG004032 and BAnTr.GP.UnG004334), and JMJ706 (BAnTr.GP.1HG007084). Indeed, of 21 AGO orthologs, 10 are differentially expressed in at least one stage or tissue comparison (Supplementary Table 7 and Supplementary Figure 7). When the log_2_FC cut-offs for differential expression are disregarded 14 AGO orthologs are significant in at least one contrast group. HvAGO18 is significantly enriched in four out of six contrast groups—most significantly (*P* = 0.0000108; log2FC + 4.17) in anthers at leptotene–zygotene compared to pre-meiosis. HvAGO18 transcript increases significantly in abundance in anthers again at pachytene–diplotene (*P* = 0.005; log2FC + 1.5) and declines slightly, although not significantly at metaphase–tetrad. HvDCL5 was significantly up regulated in anthers at leptotene–zygotene stage compared to pre-meiosis (*P* = 0.0015). HvRDR6 was significantly enriched in meiocytes when compared to whole anther tissue at leptotene–zygotene (*P* = 0.0047).

Protein-coding genes which are up-regulated in anthers at successive stages and down-regulated in meiocyte versus anther contrast groups of the same stage are largely reflective of water transport, photosynthesis, cell signaling, amino acid transport, and other metabolic processes (Supplementary Table 3). However, down-regulated DEGs in anthers at leptotene–zygotene versus pre-meiosis are more informative. The switch to prophase I in anthers, in addition to the large-scale down-regulation of lncRNAs, coincides with massive down-regulation of protein-coding genes annotated with RNA-directed DNA polymerase activity (GO:0003964; *P* < 1E-30) all of which are expressed only in anther tissues and not in germinating embryos. Several of these genes were annotated as reverse transcriptase-like genes from *copia-*type centromeric retrotransposons RE1 and TNT 1 in *Arabidopsis* and *N. tabacum* respectively (Grandbastien et al., 1989; Yamada et al., 2014). These also formed a sizeable component of the down-regulated DEGs annotated as nucleic acid binding (GO:0003676; *P* < 1E-30) and zinc binding (GO:0008270; *P* < 1E-30) proteins; 89% of both groups were also anther specific. DNA binding transcription factors (GO:0003700; *P* = 3.20E-5) and transcriptional regulation related GO terms GO:0017053 and GO:0006355 (*P* = 5.50E-5 and 2.20E-6, respectively) were also enriched in down-regulated DEGs in this contrast group.

### Many Meiotic Genes Are Expressed Before Meiosis Begins

**FIGURE 6:**
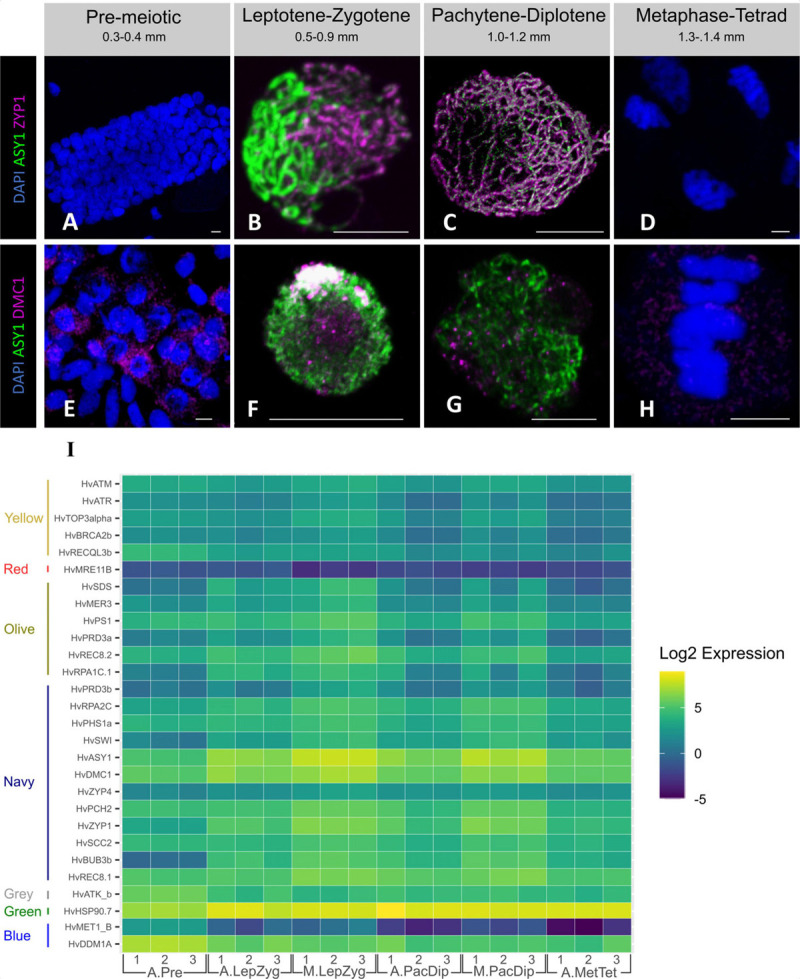
Expression of selected meiotic genes. Immuno-staining of meiotic nuclei at four developmental stages for HvZYP1 (magenta, **A–D**), and HvDMC1 (red, **E–H**) proteins. All samples were stained with anti-ASY1 antibody (green) and counterstained with DAPI (blue). **(A,E)** Pre-meiosis, **(B,F)**, leptotene—zygotene, **(C,G)**, pachytene—diplotene, **(D,H)**, anaphase I—metaphase I. Scale bar 10 μm. **(I)** Heatmap expression profile of meiotic genes with a statistically significant log fold change in at least one tissue or stage comparison. The genes are ordered and grouped by WGCNA module on the vertical axis. Genes were extracted from the total dataset, transcript counts log transformed, and plotted using ggplot2 (Wickham, 2016) in R (script available at https://github.com/BioJNO/BAnTr). The samples (3 replicates each) are A.Pre, anther pre-meiosis; A.LepZyg, anther leptotene–zygotene; A.PacDip, anther pachytene–diplotene; A.MetTet, anther metaphase I–tetrad; M.LepZyg, meiocyte leptotene–zygotene; M.PacDip, meiocyte pachytene–diplotene. The prefixes A. and M. in the sample names depict anther and meiocyte samples, respectively.

We probed pre-meiotic and meiotic cells with antibodies against two SC (ASY1 and ZYP1) proteins and one recombination (DMC1) protein. The protein products of *HvASY1* and *HvZYP1* were detected during prophase I (Figures 6B,C) but not in pre-meiotic and anaphase I cells (Figures 6A,D). HvDMC1 detection on the other hand shows a diffuse, mostly cytoplasmic, signal in pre-meiotic cells (Figure 6E). During meiosis, HvDMC1 appears as discrete foci in leptotene–diplotene nuclei (Figures 6F,G) before becoming diffuse again at metaphase I–tetrad (Figure 6H). All three genes are transcribed at pre-meiosis (Figure 6I) and belong to the navy WGCNA module showing higher transcript abundance in prophase I meiocytes (Figure 2). We then analyzed the gene-level expression of 121 known meiotic genes which were distributed among 11 different modules. While all were expressed, only 29 were differentially expressed across the samples using our experimental thresholds (Figure 6I and Supplementary Figure 6). It is noticeable that the overall level of expression of some genes, like *HvPCNA* and members of the *HSP90* family, remains high in all stages and tissues while others, like *HvMET1b*, *HvMRE11B*, *HvXRCC3*, and *HvDDM1C*, remained relatively low.

Of the 28 meiotic genes, *HvASY1*, *HvDMC1, HvZYP1*, *HvBUB3b*, *HvSDS*, *HvSWI*, *HvRPA2c*, *HvRPA1c.1*, and *HvHSP90.7* were significantly up-regulated from pre-meiosis to leptotene–zygotene. Further gene-level expression comparisons showed 19 putative meiotic genes (*HvASY1*, *HvATM*, *HvATR*, *HvBRCA2b*, *HvBUB3b*, *HvMER3*, *HvPCH2*, *HvPHS1a*, *HvPRD3a*, *HvPRD3b*, *HvPS1*, *HvREC8.1*, *HvREC8.2*, *HvRPA2C*, *HvSCC2*, *HvSWI*, *HvTOP3alpha*, *HvZYP1*, and *HvZIP4*) were significantly enriched in meiotic cells compared to anthers at the same stage (Supplementary Table 8). In comparison to our control dataset of germinating embryo samples, expression of only 6 of these meiotic genes are specific to anthers. However, while the rest are expressed in the EMB samples, 16 show lower gene expression in this tissue. The other nine genes show EMB gene expression similar to at least one anther developmental stage.

### A Large Number of Transcription Factors Are Differentially Expressed During Anther and Meiocyte Development

**FIGURE 7:**
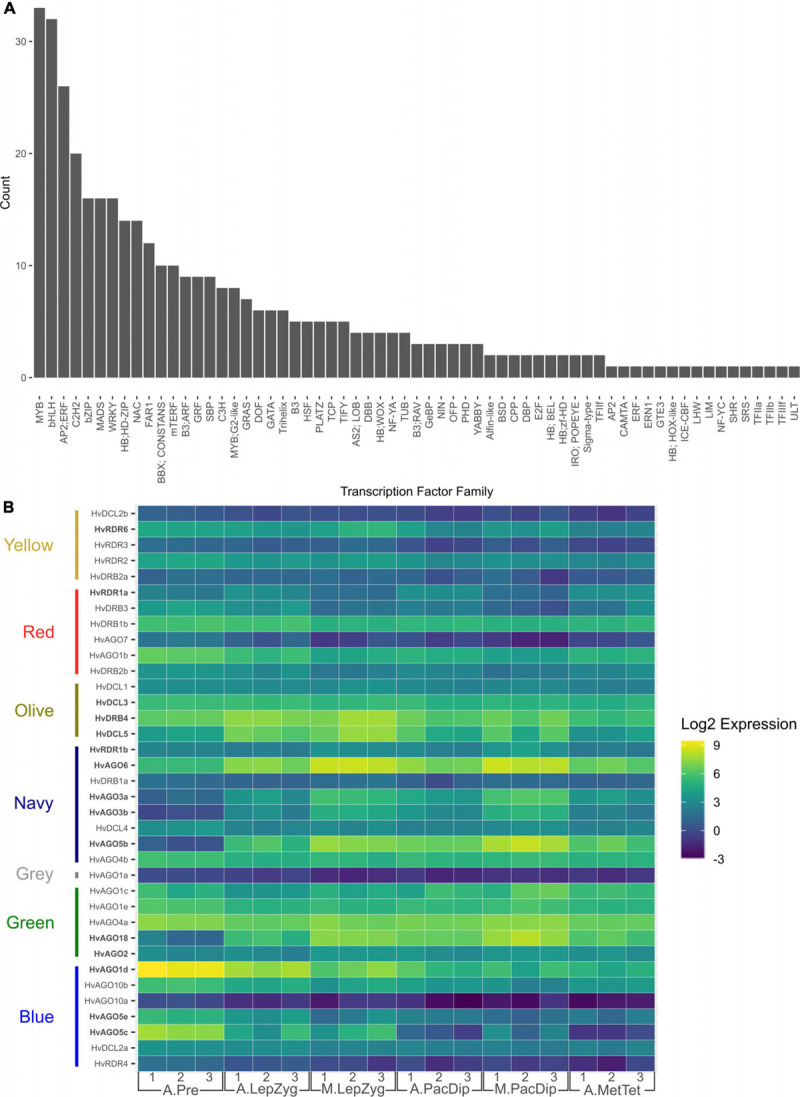
Expression of transcription factor (TF) families in anthers and meiocytes**. (A)** Total number of TFs per family that are expressed in anthers and meiocytes. **(B)** Number of differentially expressed TFs determined by comparing their transcript levels in anthers at different stages. The comparisons are: A.LepZyg-A.Pre, anther leptotene–zygotene versus anther pre-meiosis; A.PacDip-A.LepZyg, anther pachytene–diplotene versus anther leptotene–zygotene; A.MetTet-A.PacDip, anther metaphase I–tetrad versus anther pachytene–diplotene. The prefixes A. and M. in the sample names depict anther and meiocyte samples, respectively.

Anther and meiocyte transcriptomes have been determined in several plant species but the mechanisms of gene regulation in these tissues are still poorly understood. The varied gene-level expression patterns of modules generated by WGCNA analysis in this dataset could be explained by steady-state gene expression regulation, including at the transcriptional level. Using the Plant Transcription Factor (TF) Database (Pérez-Rodríguez et al., 2010), we found a total of 1,353 annotated TFs of which 382 (28.2%)—belonging to several different TF families (Supplementary Table 8)—were differentially expressed in at least one stage or tissue comparison. 140 of these were expressed in anther tissues and not in germinating embryos. The largest families in the significant log_2_FC subset are bHLH (*n* = 29) and MYB (*n* = 29) (Figure 7A). The distribution of TFs families in different expression modules was determined with the red module containing the highest number (*n* = 154; Supplementary Table 9), a module showing down-regulation in meiocytes when compared to anthers at both stages of leptotene–zygotene and pachytene–diplotene (Supplementary Figure 3). This is reflected in the significant GO enrichment of transcriptional regulation in anthers at leptotene–zygotene versus pre-meiosis. 254 (66.5% of transcription factors significant in any contrast group) showed differential expression in comparisons between meiocytes and anthers at either pachytene–diplotene or leptotene–zygotene, with 170 and 84 TFs significantly different in one or both stages, respectively (Figure 7B and Supplementary Table 10). This strong transcriptional activation, accompanied by meiocyte enrichment in E3 ubiquitin ligases for likely protein degradation, might underly the vast transcriptome reprogramming at the meiosis onset.

## Discussion

Despite technical advances, and the importance of the reproductive phase in crop breeding, the regulation of the meiotic transcriptome in plants is still poorly understood (Zhou and Pawlowski, 2014). In this study, we sought to establish the most comprehensive transcriptome of barley anthers and meiocytes spanning pre-meiosis–tetrad stages. The barley cultivar Golden Promise (GP) was chosen as it is the reference cultivar for barley transformation which is relevant for future functional studies. In addition, we recently sequenced its genome (Schreiber et al., 2020), which will assist with interpretation of the transcriptome data, and developed a TILLING population using ethyl methanesulfonate (Schreiber et al., 2019). We used immuno-cytology to carefully stage all anther and meiocyte samples and analyzed a combination of Illumina and PacBio reads using the most up to date bioinformatic pipeline. This allowed us to identify novel transcripts and study transcriptome dynamics throughout meiosis.

### lncRNAs Are Enriched in Pre-meiotic Anthers but Not in Meiocytes

The number of DEGs in staged anther tissues declines from its highest level between leptotene–zygotene and pre-meiosis with the largest down-regulation of genes of any comparison. This supports previous studies in *Arabidopsis* (Chen et al., 2010; Yang et al., 2011), maize (Dukowic-Schulze et al., 2014b; Nelms and Walbot, 2019) and sunflower (Flórez-Zapata et al., 2014, 2016) showing large scale transcriptional re-organization at meiosis entry. A strikingly large component of differentially expressed genes at this transition were lncRNAs—98% of which were expressed in anthers but not in germinating embryos. High expression of lncRNAs in plant reproductive tissues has been described in both sunflower (Flórez-Zapata et al., 2016) and in maize (Li et al., 2014). Flórez-Zapata et al. (2016) reported large scale differential expression of lncRNAs in sunflower meiocytes when contrasted with somatic tissues; and between meiocytes in comparison of genotypes exhibiting significantly different recombination rates. Our results indicate that anther specific lncRNAs may play an important role in prophase I entry in barley. However, our results also indicate that expression of many of these lncRNAs is down-regulated during the transition to prophase I and in meiocytes when compared to whole anther tissues—indicating that their most important functional roles may be prior to meiosis or as pre-cursors to meiotic small RNAs (sRNAs).

Rapid evolution of lncRNAs is well documented; however, in most cases function is conserved across large evolutionary distances (Ulitsky, 2016). This is reflected in the low proportion of BAnTr lncRNAs which align to published lncRNA database sequences. LncRNAs are emerging as key plant development regulators (Ariel et al., 2015; Shafiq et al., 2016) and their role in animal and plant sexual reproduction is now well established (reviewed in Golicz et al., 2018), including transcriptional gene silencing (Wierzbicki et al., 2008), nucleosome positioning (Garcia et al., 2010), chromosome looping (Ariel et al., 2014), and pairing of homologous chromosomes (Ding et al., 2012). LncRNAs are also precursors of phasiRNAs—cleaved double-stranded RNAs either 21 or 24 nt in length. Both 21 and 24 nt phasiRNAs are associated with male reproductive development in angiosperms, the former predominantly and the latter exclusively. We have identified 568 and 553 PHAS transcripts with predicted miR2118 and miR2275 recognition sites, respectively, and only 53 lncRNAs contain recognition sites for both miRNAs. These numbers are larger than previously reported for maize anthers using small RNA sequencing in maize (Zhai et al., 2015). RDR6 is involved in processing both classes, converting single-stranded precursor RNA into double-stranded RNA (dsRNA) following AGO-catalyzed cleavage (Song et al., 2012b). DCL4 and DCL5 then cuts this dsRNA in regular intervals producing 21 and 24 nt phasiRNAs, respectively (Song et al., 2012a; Zhang M. et al., 2020). Both RDR6 and DCL5 are present in co-expression networks enriched in meiocytes in the BAnTr dataset. RDR6 showed significant enrichment at leptotene–zygotene in meiocytes compared to anthers and DCL5 was up-regulated in anthers at leptotene–zygotene compared to pre-meiosis (Supplementary Figure 7 and Supplementary Table 7). DCL5 has been shown to generate 24-nt phasiRNAs, which in turn silence the 24-PHAS loci in cis by CHH-type DNA methylation during meiotic anther development in maize (Zhang M. et al., 2020). This may reflect the importance of 24-nt phasiRNAs in prophase I. Several AGO genes also showed significant differential expression. HvAGO18, orthologous to AGO18 which is a suggested 24-nt phasiRNAs binding partner (Zhai et al., 2015; Fei et al., 2016), is significantly enriched in four out of six contrast groups—most significantly (*P* = 0.0000108; log2FC + 4.17) in anthers at leptotene–zygotene compared to pre-meiosis. HvAGO18 transcript increases significantly in abundance in anthers again at pachytene–diplotene (*P* = 0.005; log2FC + 1.5) and declines slightly, although not significantly at metaphase–tetrad. HvAGO18—and several other AGO orthologs—are enriched in meiocytes compared to anthers in both staged prophase I sample groups. phasiRNAs have been shown to be enriched in anthers at different meiotic stages in maize (Zhai et al., 2015) and rice (Komiya et al., 2014; Fei et al., 2016) and shown to be present in most angiosperms (Xia et al., 2019). Deep sequencing technologies have enabled global analysis of meiotic sRNA in sunflower showing sequence similarity to 40% of lncRNAs (Flórez-Zapata et al., 2016). In *Arabidopsis*, Huang et al. (2019) revealed different meiotic and mitotic sRNA landscapes and found that meiocyte-specific sRNAs (ms-sRNAs) are significantly enriched in genic regions contrary to somatic small interfering RNAs that are enriched in intergenic regions. A high proportion of these ms-sRNAs (69%) were found to be DSB-dependent (Huang et al., 2019). A detailed analysis of genome-wide distribution of PHAS loci and their expression throughout anther development in wheat and barley has uncovered a new 24 nt pre-meiotic class (Bélanger et al., 2020). Further analyses will be needed to fully elucidate the relationship of lncRNAs described in our study and sRNAs.

#### Methylation, Demethylation, and AGO Genes

sRNAs biogenesis and function require several players including RNA polymerases (Pol), Dicer-like (DCL) proteins, double-stranded RNA-binding (DRB) proteins, RNA-directed RNA polymerases (RDRs) and AGO proteins (Borges and Martienssen, 2015; Yu et al., 2018). We checked the expression dynamics of sRNA biogenesis and AGO genes, for which protein products were detected in our recent anther proteomic study (Lewandowska et al., 2019), and found the expression of 10 out of 21 AGO genes changed significantly during barley anther and meiocyte development (Supplementary Figure 7). This gene expression profile highlights the importance of sRNA biogenesis and their guiding AGO proteins during meiosis. A new study characterized the components of phasiRNA biogenesis pathway in wheat and barley and found that most AGO genes were expressed in anthers of both species (Bélanger et al., 2020). Expression profiles of different sRNA biogenesis compounds, including AGOs we found in this study, were similar to those reported by Bélanger et al. (2020). These authors correlated the expression pattern of different gene family members with those of pre-meiotic 21 and 24 nt and meiotic 24 nt phasiRNA classes delineating their potential role in different anther developmental stages. However, further analysis of these pathways and their target loci is needed to fully describe their role in meiotic development. One such function has been demonstrated in a recent study by Walker et al. (2018) showing that *de novo* RNA-directed DNA methylation (RdDM) induces cell-lineage-specific epigenetic marks regulating meiotic gene expression in *Arabidopsis*.

mRNA methylation is emerging as another important level of meiotic gene regulation. The N^6^-methyladenosine (m^6^A) modification was shown to be associated with mRNA translatability in yeast (Bodi et al., 2015) and *Xenopus* (Qi et al., 2016). More recently, Bushkin et al. (2019) showed that m^6^A methylation at the 3′ UTR of the mRNA encoding Rme1p, a transcriptional repressor of meiosis, results in its degradation allowing meiosis initiation in yeast. Current epitranscriptome methods necessitate a large amount of mRNA making it difficult to implement in plant meiocytes. Instead, we determined transcript levels of known plant m^6^A pathway genes (Yue et al., 2019), for which protein products were detected in our anther proteomic study (Lewandowska et al., 2019). The expression of these genes was relatively low and stable throughout anther and meiocyte development. However, the translation status and potential function in these tissues remains to be elucidated. It will be interesting to compare the expression profiles of these enzymes in different plant species. Indeed, Liu J. et al. (2020) profiled m^6^A and N^6^,2′-O-dimethyladenosine (m^6^Am) across human and mouse tissues and suggested that the difference in these epitranscriptomic marks is greater between species than tissue types.

### Significant Post-transcriptional and Post-translational Regulation During Prophase I

Comparison of meiocytes and anthers at leptotene–zygotene presented the greatest contrast in protein-coding gene expression. GO enrichment analysis of DEGs in this contrast group suggests heightened importance of ubiquitination, RNA modification, and histone demethylation in prophase I regulation.

#### Post-transcriptional RNA Modification Through PPR Proteins

Significant enrichment of the RNA modification GO term in meiocytes compared to anthers at leptotene–zygotene reflected several up-regulated DEGs annotated as PPR proteins. PPR proteins are found in all eukaryotes but are remarkably abundant in land plants, indicative of massive expansion during land plant evolution (Barkan and Small, 2014; Gutmann et al., 2020). Through sequence-specific binding, PPR proteins can mediate RNA folding, splicing, degradation, cleavage, and editing (Barkan and Small, 2014). PPR proteins are associated with organelles, deleterious mutations in which lead to defects in photosynthesis or oxidative phosphorylation (Barkan and Small, 2014). Dukowic-Schulze et al. (2014b) reported up-regulation of 24 mitochondrial genes in maize meiocytes compared to whole anther tissues and seedlings, arguing that this indicated high energy demand concomitant with chromosome movement in early prophase I. Further, Yuan et al. (2018) and Nelms and Walbot (2019) also reported significant GO enrichment of various mitochondrial processes in developing germinal cells. Up-regulation of PPR proteins in early barley prophase I may support this, indicating a general increase in mitochondrial activity in step with a detectable spike in PPR protein mediated RNA regulation.

#### Ubiquitin Ligases and Deubiquitinating Enzymes

E3 ubiquitin ligases are an important regulatory component during meiosis in many organisms (Okamato et al., 2012; Mohammad et al., 2018). E3 ligases interact with target proteins to facilitate, directly or indirectly, modification of the substrate with ubiquitin—conferring substrate specificity to the ubiquitination cascade (Iconomou and Saunders, 2016). Several E3 ligases, and multi-subunit E3 ligase components, have been described with significant roles in meiosis in *Arabidopsis* (Yang et al., 1999, 2006; Wang and Yang, 2006), rice (He et al., 2016; Zhang F. et al., 2017), and wheat (Li et al., 2006; Hong et al., 2013). Further, enrichment of E3 ligases in pre-meiotic pollen mother cells has been reported in both rice (Tang et al., 2010) and maize (Yuan et al., 2018). Our findings suggest that ubiquitination through E3 ligase activity is of continued importance throughout prophase I. E3 ligase genes up-regulated in meiocytes include an ASK1 ortholog, an *Arabidopsis* S-phase kinase-associated protein 1 (SKP1) which interacts with Cullin and F-box proteins to form the SKP-Cullin-F-box (SCF) E3 ligase complex (Yang et al., 1999). SKP1-like proteins are functionally conserved in plants, with the wheat ASK1 equivalent, TSK1, able to partially rescue fertility in *Arabidopsis ask1* mutants (Li et al., 2006). ASK1 was identified as a negative regulator of male recombination, essential for the proper release of chromatin from the nuclear membrane (Wang and Yang, 2006; Yang et al., 2006). Several F-box proteins, putatively interacting in the SCF E3 ubiquitin ligase complex, also displayed differential expression throughout prophase I. In rice, mutations in two F-box proteins interacting with the ASK1 equivalent OSK1—MOF and ZYGO1—have also been shown to result in male sterility (He et al., 2016; Zhang F. et al., 2017). Several genes annotated as SINA E3 ligases also displayed differential expression during prophase I. A recent study in *Drosophila melanogaster* females identified a SINA E3 ligase which regulated both assembly and disassembly of the SC, preventing aberrant polymerization and polycomplex formation of SC components (Hughes et al., 2019). Although no such function of SINA E3 ligase is so far reported in plants, the E3 ligases highlighted in this work are attractive candidates to investigate a similar role for these proteins in regulating SC formation in barley. DUBs, particularly those targeting lys48 ubiquitin chains, are also differentially expressed throughout prophase I. DUBs facilitate removal of ubiquitin from proteins; in this way they reverse ubiquitination mediated by E3 ligases. Lys48 is one of seven lysine residues through which ubiquitin can form covalent C-terminally linked chains (Kulathu and Komander, 2012; López-Mosqueda and Dikic, 2014). Although targeting for proteasomal degradation is the canonical function of ubiquitination, first described by Ciehanover et al. (1978), many other effects of this modification have been observed such as recruitment of binding partners (Huang and D’Andrea, 2010), activation (Xu et al., 2009), or nuclear uptake (Plafker et al., 2004). The diversity of substrate fates is derived from diversity in chain topology (Komander and Rape, 2012). Simultaneous up-regulation of E3 ubiquitin ligase and DUB expression may represent both very tight control of substrate modification, where ligase and DUB build and remove the same chain topology, respectively, and specific reduction in lys48 ubiquitination while alternative chain linkage types are enriched, where they do not.

### Expression of Meiotic Genes Does Not Always Reflect the Timing of Their Functional Roles in Meiocytes

Using phylogenomic analysis, we compiled a large inventory of barley orthologs of known meiotic genes. We determined their relative expression levels and found them to be distributed in 11 different expression modules. In total, 24% of selected meiotic genes were differentially expressed during anther and meiocyte development.

Like previous studies, our transcriptomic data provided evidence that many known meiotic genes are transcribed prior to prophase I. However, the timing of their translation is still not fully resolved due to limited availability of antibodies (this work), issues related to sample staging, and proteomic resolution (Zhang et al., 2014; Yuan et al., 2018). Genome-wide ribosome profiling could be achieved by using transgenic tools like RiboTag/RNA-seq (Lesiak et al., 2015). Such analysis was performed in mice revealing alteration of maternal mRNA translation in oocytes at meiotic re-entry (Luong et al., 2020).

### Expression of Transcription Factors Changes Throughout Anther Stages

To decipher the mechanisms of meiotic transcriptome regulation, we analyzed our transcriptomic data for transcriptional regulation. The involvement of many transcription factors (TFs) in transcriptome reprogramming during pre-meiotic anther differentiation has been shown in maize (Zhang et al., 2014). This study was recently expanded by analyzing TFs expression at tissue level using microdissection (Yuan et al., 2018). However, both studies used microarrays, a closed technology, that may underestimate the number of TFs. We found a total of 1,353 annotated TFs (63 families) of which 28.2% were differentially expressed in at least one stage or tissue comparison. In agreement with previous maize studies, our data also reveal major TFs expression changes occurring at and beyond mitotic–meiotic transition in whole anthers. However, no such changes were observed in meiocytes. Beside these global analyses, functional studies of TFs are still lacking to fully understand meiotic gene regulation. More recently, *TBP-ASSOCIATED FACTOR 4b* (*TAF4b*), encoding a subunit of the RNA polymerase II general transcription factor TFIID, was shown to be enriched in meiocytes and to control transcription of genes involved in the meiotic cell cycle and recombination in *Arabidopsis* (Lawrence et al., 2019). In our study, a single barley TAF4b homolog gene (BAnTr.GP.6HG009004) was found in the green module showing increasing expression (*P* < 0.01) throughout anther and meiocyte stages, though below our threshold of log_2_FC > 1 in all comparisons.

### Alternative Splicing

Although alternative splicing (AS) is known to increase the genome coding capacity, its activity during meiosis has been studied as far as we are aware only in mouse testis (Schmid et al., 2013) and yeast (Kuang et al., 2017). Intron retention events were found to be enriched in both mouse (Naro et al., 2017) and yeast meiocytes (Kuang et al., 2017). They were also enriched during *Arabidopsis* flower development (Wang et al., 2014). Here, GO enrichment analysis showed enrichment of terms that are connected to alternative splicing. This contained genes involved in forming the spliceosome or spliceosome-associated non-snRNP proteins. Contrary to our expectations we only identified two meiotic genes as significantly differentially alternatively spliced. In future we would like to extend our potential meiotic gene list as the 121 genes are only a small subset and need to be extended to potential orthologous genes identified in studies from mice, yeast, and *Arabidopsis*. We also might be able to extend the list and add barley specific meiotic genes through this dataset.

## Conclusion and Perspectives

Our analysis of barley anther and meiocyte transcriptomes provides evidence of a multi-faceted regulatory network orchestrating meiotic gene regulation. Sequencing of sRNA and DNA methylation, as done in maize by Dukowic-Schulze et al. (2016), in anther and meiocyte at pre-meiosis to leptotene–zygotene stages with the strongest transcriptomic changes will enhance the full picture of these interconnecting gene regulation mechanisms. Newly developed technologies, like single-cell RNA-seq (Denyer et al., 2019) and spatial transcriptomics (Giacomello et al., 2017), could increase the resolution of anther and meiocyte transcriptomes even at sub-cellular level. Nascent transcript sequencing by NET-seq (Churchman and Weissman, 2011), GRO-seq (Hetzel et al., 2016) or Neu-seq (Szabo et al., 2020) and ribosome profiling by RiboTag-seq (Lesiak et al., 2015), for example, could be used to directly evaluate transcriptional and translational activities throughout anther and meiocyte development.

The combination of immuno-cytological staging and RNA-seq data has allowed us to build a robust time-resolved barley anther and meiocyte transcriptomic dataset. We detected large scale down-regulation of lncRNAs at meiosis entry in anthers and enrichment of DAS at early prophase I. In addition to changes in expression, we also revealed the diversity of transcription factors accompanied by several other post-transcriptional and post-translational regulatory networks. Our data will be informative for research in anther and meiocyte development in other plant species. In addition, it has already contributed to building a first barley reference transcript dataset (BaRTv1) (Rapazote-Flores et al., 2019), to constructing a reference database for proteomic studies of staged anthers (D. Lewandowska et al., unpublished) and for annotation of barley reference genome and pangenome sequences (Monat et al., 2019; Jayakodi et al., unpublished).

## Data Availability Statement

The datasets presented in this study can be found in online repositories. The names of the repository/repositories and accession number(s) can be found below: https://www.ncbi.nlm.nih.gov/, PRJNA558196; https://www.ncbi.nlm.nih.gov/, PRJNA593943; https://figshare.com/, https://doi.org/10.6084/m9.figshare.11974182, https://figshare.com/, http://doi.org/10.6084/m9.figshare.12136773, https://figshare.com/, http://doi.org/10.6084/m9.figshare.12136773, https://figshare.com/, http://doi.org/10.6084/m9.figshare.12136773.

## Author Contributions

AB, DL, IC, MS, LR, and RW: experimental design. NM: plant growth. AB, DL, IC, MA, MM, MS, and NM: collection of anthers. IC: collection of meiocytes and immuno-cytology. AB, PH, and JM: RNA extraction, library prep, and QC. MS: transcriptome assembly. AB: lead manuscript preparation. AB, DL, JO, IC, MS, and RW: data analysis and manuscript preparation. All authors contributed to the article and approved the submitted version.

## Conflict of Interest

The authors declare that the research was conducted in the absence of any commercial or financial relationships that could be construed as a potential conflict of interest.

## Footnotes

1 https://github.com/PacificBiosciences/IsoSeq3

2 https://github.com/TransDecoder/TransDecoder

## Supplementary Material

Supplementary Figure 1 3D MDS plot of sample transcript expression.

Supplementary Figure 2 3D RNAseq overview and long non-coding RNA (lncRNA) categories.

Supplementary Figure 3 WGNCA co-expression modules.

Supplementary Figure 4 Eigengene network analysis.

Supplementary Figure 5 WGCNA GO enrichment analysis.

Supplementary Figure 6 Heat map of meiotic gene expression.

Supplementary Figure 7 Heat map sRNA gene silencing protein expression.

Supplementary Table 1 Raw read counts.

Supplementary Table 2 Number of genes per WGCNA modules.

Supplementary Table 3 GO enrichment analysis results.

Supplementary Table 4 Differential expression analysis.

Supplementary Table 5 lncRNA database BLAST hits by classification.

Supplementary Table 6 Differentially Expressed E3 Ubiquitin ligases.

Supplementary Table 7 Expression of sRNA gene silencing orthologs.

Supplementary Table 8 Differential expression analysis results for Meiotic genes.

Supplementary Table 9 Distribution of transcription factor families in different WGCNA modules.

Supplementary Table 10 Transcription factor families in different comparisons.

Supplementary Method 1 Immunocytology.

Supplementary Method 2 Isolation of meiocytes.

Supplementary Method 3 RNA extraction and sequencing.

Supplementary Method 4 Exact parameters of Illumina read mapping.

Supplementary Video 1 Isolated fresh meiocytes bag at Leptotene/zygotene.

Supplementary Video 2 Isolated fresh meiocytes bag at pachytene/diplotene.

Supplementary File 1 Assembled BAnTr Transcriptome fasta file. AntherTranscriptomeBAnTr.fasta at http://doi.org/10.6084/m9.figshare.12136773.

Supplementary File 2 Padded BAnTr Transcriptome fasta file. AntherTranscriptomeBAnTrPadded.fasta at http://doi.org/10.6084/m9.figshare.12136773.

Supplementary File 3 BAnTr Proteome fasta file. AntherProteomeBAnTr.fasta at http://doi.org/10.6084/m9.figshare.12136773.

## Gene Glossary

HSP90.7 HEAT SHOCK PROTEIN 90.7

PTB1a POLYPYRIMIDINE TRACT BINDING PROTEIN 1a

HEI10 HUMAN ENHANCER OF INVASION 10

NEDD8 NEURAL PRECURSOR CELL EXPRESSED DEVELOPMENTALLY DOWN-REGULATED PROTEIN 8

KDM3 LYSINE SPECIFIC DEMETHYLASE 3

PKDM9 PUTATIVE LYSINE SPECIFIC DEMETHYLASE 9

DCL5 DICER-LIKE 5

RDR6 RNA DEPENDENT RNA POLYMERASE 6

ASY1 ASYNAPTIC 1

ZYP1 MOLECULAR ZIPPER 1-LIKE PROTEIN

DMC1 DISRUPTED MEIOTIC CDNA 1

PCNA PROLIFERATING CELL NUCLEAR ANTIGEN

MET1B DNA (CYTOSINE-5)-METHYLTRANSFERASE 1B

MRE11B MEIOTIC RECOMBINATION 11 HOMOLOG B

XRCC3 X-RAY REPAIR CROSS COMPLEMENTING 3

DDM1C DECREASED DNA METHYLATION 1C

BUB3B BUDDING UNINHIBITED BY BENZIMIDAZOLES 3B

SDS SOLO DANCERS

SWI SWITCH 1/DYAD

RPA2c REPLICATION PROTEIN A2C

RPA1c.1 REPLICATION PROTEIN A1C.1
