# Supplementary material for: Barley Anther and Meiocyte Transcriptome Dynamics in Meiotic Prophase I
Source: Front Plant Sci. 2021 Jan 12;11:619404. doi: 10.3389/fpls.2020.619404 (PMC7835676; doi:10.3389/fpls.2020.619404)
Supplement: Supplementary Figure 1 — 3D MDS plot of sample transcript expression. [file Data_Sheet_1.PDF]

Supplementary Material

1 Supplementary methods

Supplementary Method 1: Immunocytology.

The primary antibody solution consisted of anti-TaASY1 (rabbit), anti-HvZYP1 (rat) and anti-HvDMC1 (guinea pig) at 1:1000, 1:500 and 1:200, respectively in 1x PBS blocking buffer (Colas et al, 2017 & 2019). Primary antibodies were added on the slide, incubated in a wet chamber for 30 minutes at room temperature followed by up to 36 hours at 4 – 6°C. Slides were warmed for 1 hour at room temperature before washing for 15 minutes in 1x PBS and incubating for 90 minutes at room temperature in the secondary antibody solution consisting of a mixture of anti-rabbit Alexa Fluor® (488) with anti-rat Alexa Fluor® 568 and/or anti-guinea pig Alexa Fluor® 568 (Invitrogen, Thermo Fisher Scientific) diluted in 1x PBS (1:600). Slides were washed for 15 minutes in 1x PBS and mounted in Vectashield® containing DAPI (H-2000, Vectorlabs). 3D Confocal stack images (512x512, 12 bits) were acquired with LSM-Zeiss 710 using laser light (405, 488, 561 nm) sequentially (4 lines average, bidirectional, optimal section). Stack images were processed using Imaris 9.5.1 (Bitplane) after image processing using laser confocal deconvolution and light gaussian filter.

Colas, I., Darrier, B., Arrieta, M., Mittmann, S.U., Ramsay, L., Sourdille, P., and Waugh, R. (2017). Observation of Extensive Chromosome Axis Remodeling during the "Diffuse-Phase" of Meiosis in Large Genome Cereals. *Frontiers in Plant Science* **8**:1235.

Colas, I., Barakate, A., Macaulay, M., Schreiber, M., Stephens, J., Vivera, S., Halpin, C., Waugh, R., and Ramsay, L. (2019). *desynaptic5* carries a spontaneous semi-dominant mutation affecting *Disrupted Meiotic cDNA 1* (DMC1) in barley. *Journal of Experimental Botany* **70**: 2683–2698.

Supplementary Method 2: Isolation of meiocytes

To isolate meiocytes, 30 to 50 anthers of the same length and meiotic stage based on acetocarmine staining were collected in a 1.5 ml Eppendorf tube containing 0.01 M citrate buffer pH 4.5 and kept on ice. Ten random anthers were transferred into a new tube with 1x PBS for further staging with specific antibodies. A maximum of 5 anthers were transferred onto a cavity slide with 5–10 µl of cold citrate buffer. The slide was placed on a cooling pack under the stereomicroscope for dissection.

After removing their tips with insulin needles, anthers were tapped gently to release the meiocytes bag. Using RNase-free 10 µl tips, released meiocyte clusters were transferred into a new 0.5 ml Eppendorf tube containing 10 µl of citrate buffer (with added RNAse Inhibitor) and anther tissue was discarded. When all the anthers were processed, an aliquot of 10 µl was transferred onto a slide and the sample was mounted in Vectashield® containing DAPI (H-2000, Vectorlabs) to check the purity of the meiocyte clusters (Video S1 and S2). The remaining meiocytes were topped with 50 µl TRIzol® and stored in the fridge until extraction.

Supplementary Method 3: RNA extraction and sequencing

Total RNA was extracted from anthers, isolated meiocytes and EMB using TRIzol® PlusRNA Purification Kit (Thermo Fisher Scientific) following the manufacturer's instructions. Total RNA was quality checked using a Bioanalyzer 2100 (Agilent). Their RNA integrity numbers (RIN) were in the range of 5.8 – 7.3 and 7.3 – 9.7 for meiocyte and anther samples, respectively. RNA-seq libraries were constructed using TruSeq mRNA Sample Preparation kit (Illumina) as recommended by the manufacturer. Libraries were quality checked using a Bioanalyzer 2100 (Agilent) and quantified by both qPCR (KAPA Library Quantification Kit, KAPA Biosystems) and using a Qubit fluorometer. RNA-seq was performed on a NextSeq 550 (Illumina) using recommended procedures, with all 6 anther and meiocyte libraries multiplexed on a single run (2x 75 bp, high-output). Total RNA samples from barley EMB were sequenced (Illumina PE150) by Novogene (HK) Company Limited, Hong Kong. Fastq files were used for downstream analysis.

Single molecule sequencing was performed at Earlham Institute, Norwich, UK using the PacBio Iso-seq method. Two RNA samples were prepared by mixing equal amounts of total RNA representing four or two different stages of isolated anthers or meiocytes, respectively. Anthers and meiocytes full-length cDNA libraries were prepared using 2 µg of total RNA and TeloPrime Full-length cDNA Amplification kit (TATAA Biocenter) according to the manufacturer's instructions. PCR Optimisation was carried out on the two cDNA samples to determine the required number of cycles per individual sample. The final extension time was increased from 5 minutes to 7 minutes compared to the Teloprime method (Lexogen GmbH, Vienna, Austria) in order to ensure the generation of full-length PCR products, and each sample required between 16–18 PCR cycles respectively to generate sufficient cDNA for SMRTbell library preparation.

A third total RNA sample was extracted from a mixture of isolated anthers ranging from 0.3 to 1.2 mm in length. The corresponding full-length cDNA library was then generated using 1 µg total RNA and the SMARTer PCR cDNA synthesis kit (Clontech, Takara Bio Inc., Shiga, Japan) following PacBio recommendations set out in the Iso-Seq method. PCR optimisation was carried out on the full-length cDNA using the KAPA HiFi PCR kit (Kapa Biosystems, Boston USA) and 12 cycles was sufficient to generate the material required for SMRTbell library preparation. The libraries were then completed following PacBio recommendations in the Iso-Seq method.

Each cDNA sample was bead cleaned with AMPure PB beads post PCR in preparation for SMRTbell library construction. SMRTbell library construction was completed following PacBio recommendations. The cDNA libraries generated were quality checked using a Qubit Fluorometer 3.0 (Invitrogen) and sized using the Bioanalyzer HS DNA chip (Agilent Technologies, Inc.). The loading calculations for sequencing were completed using the PacBio SMRTlink Binding Calculator v5.0.0.6236. The sequencing primer from the SMRTbell Template Prep Kit 1.0-SPv3 was annealed to the adapter sequence of the libraries. Each library was bound to the sequencing polymerase with the Sequel Binding Kit v2.0 and the complex formed was then bound to Magbeads in preparation for sequencing using the MagBead Kit v2. Calculations for primer and polymerase binding ratios were kept at default values. Sequencing Control v2.0 was spiked into each library at ~1% prior to sequencing. The libraries were prepared for sequencing using the PacBio recommended instructions laid out in the binding calculator. The sequencing chemistry used to sequence all libraries was Sequel Sequencing Plate v2.1 and the Instrument Control Software version was v5.0.0.12545. The libraries were sequenced on the Sequel Instrument v1, using 1 SMRTcell v2.1 per library. All libraries had 600-minute movies, 120 minutes of immobilisation time, and 90 minutes pre-extension time.

Supplementary Method 4: Exact parameters of Illumina read mapping.

The first round of read mapping was done using the following parameters: --runThreadN 16 --outBAMsortingThreadN 16 --outSAMprimaryFlag AllBestScore --outSAMstrandField intronMotif --outSAMtype BAM SortedByCoordinate --outFilterType BySJout --outFilterMultimapNmax 15 --outFilterMismatchNoverReadLmax 0.02 --outFilterMatchNminOverLread 0.98 --outFilterIntronMotifs RemoveNoncanonical --outSJfilterOverhangMin -1 10 10 10 --outSJfilterCountUniqueMin -1 5 5 5 --outSJfilterCountTotalMin -1 10 10 10 --alignIntronMin 60 --

alignIntronMax 15000 --alignMatesGapMax 2000 --alignSJoverhangMin 7 --alignSJDBoverhangMin 7 --alignTranscriptsPerReadNmax 30000 --alignEndsType EndToEnd --alignSoftClipAtReferenceEnds No

The 22 splice junction files from the individual read mappings were used for generating the second genome index, using --jsdbFileChrStartEnd. All reads were mapped again using the second genome index, with the following parameters changed in comparison to the first sequencing run: --outSJfilterOverhangMin -1 7 7 7 --alignSJoverhangMin 5 --alignSJDBoverhangMin 5

The resulting bam files were then used for the building of a reference based transcriptome using Stringtie (Pertea et al. 2015) [-m 150 -a 5 -j 1 -f 0 -u -c 5] and scallop (Shao and Kingsford 2017) [--min_flank_length 5 --min_splice_bundary_hits 1 --min_transcript_length_base 150 --min_transcript_coverage 5 --min_single_exon_coverage 5]. The resulting 22 stringtie.gtf files were merged using stringtie --merge -f 0 -T 0.1 into one stringtie output file. The same was done for the 22 scallop.gtf files.

2 Supplementary Data

2.1 Supplementary Files

Supplementary File 1 Assembled BAnTr Transcriptome fasta file.

AntherTranscriptomeBAnTr.fasta at http://doi.org/10.6084/m9.figshare.12136773

Supplementary File 2 Padded BAnTr Transcriptome fasta file.

AntherTranscriptomeBAnTrPadded.fasta at http://doi.org/10.6084/m9.figshare.12136773

Supplementary File 3 BAnTr Proteome fasta file.

AntherProteomeBAnTr.fasta at http://doi.org/10.6084/m9.figshare.12136773

2.2 Supplementary Videos

Supplementary Video 1 Isolated fresh meiocytes bag at Leptotene/zygotene.

Supplementary Video 2 Isolated fresh meiocytes bag at pachytene/diplotene.

3 Supplementary Figures and Tables

3.1 Supplementary Figures

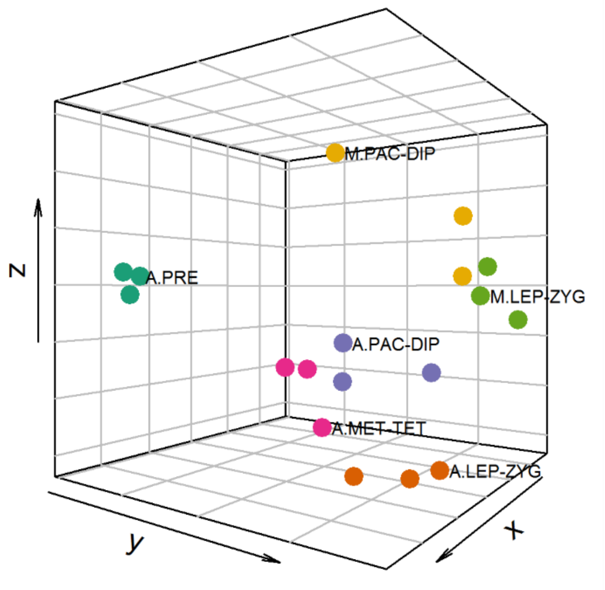

Supplementary Figure 1: Sample clustering analysis by 3D multidimensional scaling plot. A.PRE, anther pre-meiosis; A.LEP-ZYG, anther leptotene–zygotene; A.PAC-DIP, anther pachytene–diplotene; A.MET-TET, anther metaphase I–tetrad; M.LEP-ZYG, meiocyte leptotene–zygotene; M.PAC-DIP, meiocyte pachytene–diplotene. The prefixes A. and M. depict anther and meiocyte samples, respectively.

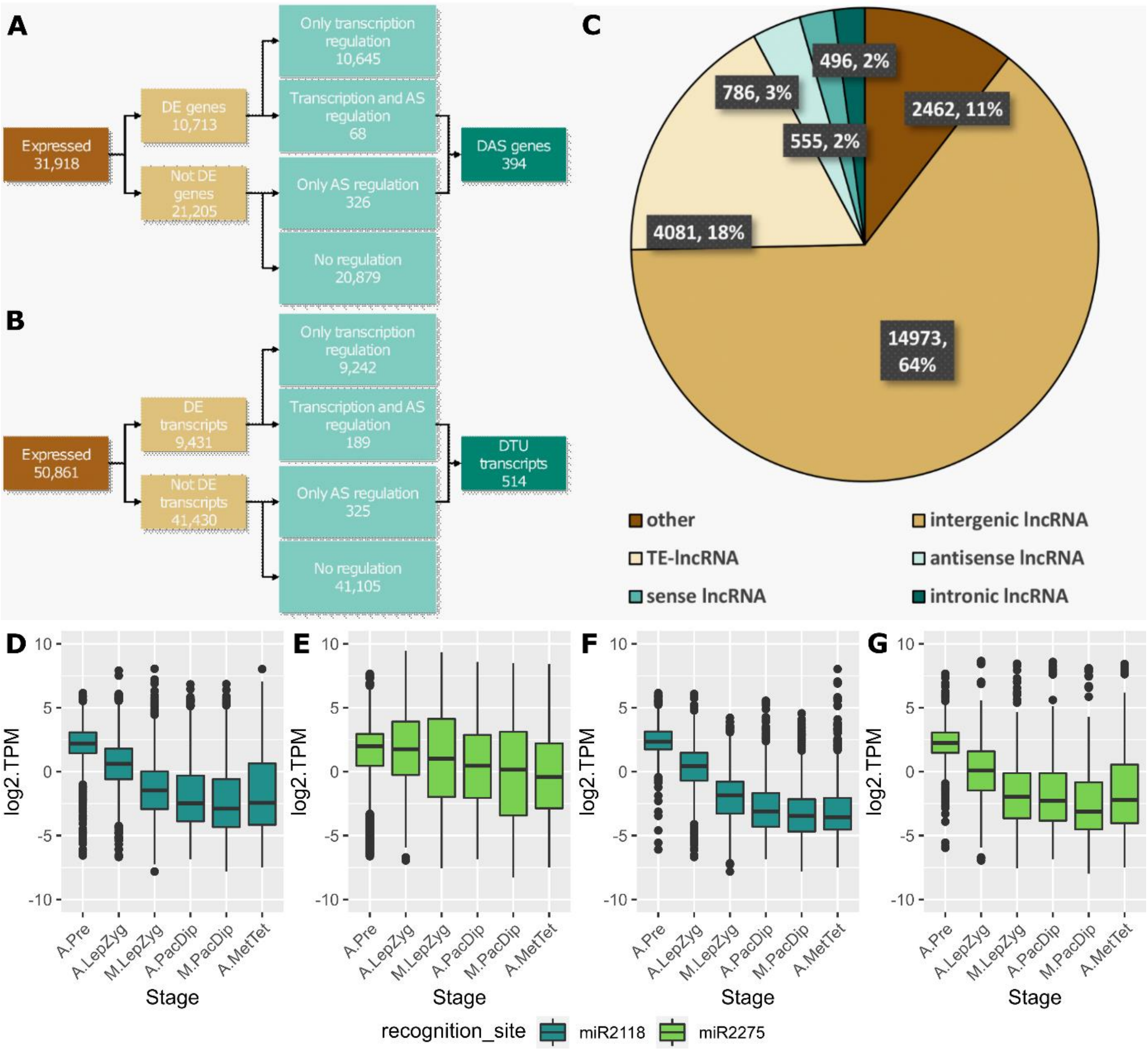

Supplementary Figure 2: Overview from the 3D RNA-seq results and long non-coding RNA (lncRNA) categories. **A**) Differential expressed genes and differential alternative spliced genes. **B**) Differential expressed transcripts and differential transcript usage transcripts. DE, differential expressed; DAS, differential alternative spliced; DTU, differential transcript usage; AS, alternative splicing. C) Proportion of different lncRNA categories. Box plots of normalised expression (log2 TPM) of all putative miR2118 (D) and miR2275 (E) targets and differentially expressed putative miR2118 (F) and miR2275 (G) targets at each stage. The samples (3 replicates each) are A.Pre, anther pre-meiosis; A.LepZyg, anther leptotene–zygotene; M.LepZyg, meiocyte leptotene–zygotene; A.PacDip, anther pachytene–diplotene; M.PacDip, meiocyte pachytene–diplotene; A.MetTet, anther metaphase I–tetrad. The prefixes A. and M. in the sample names depict anther and meiocyte samples, respectively.

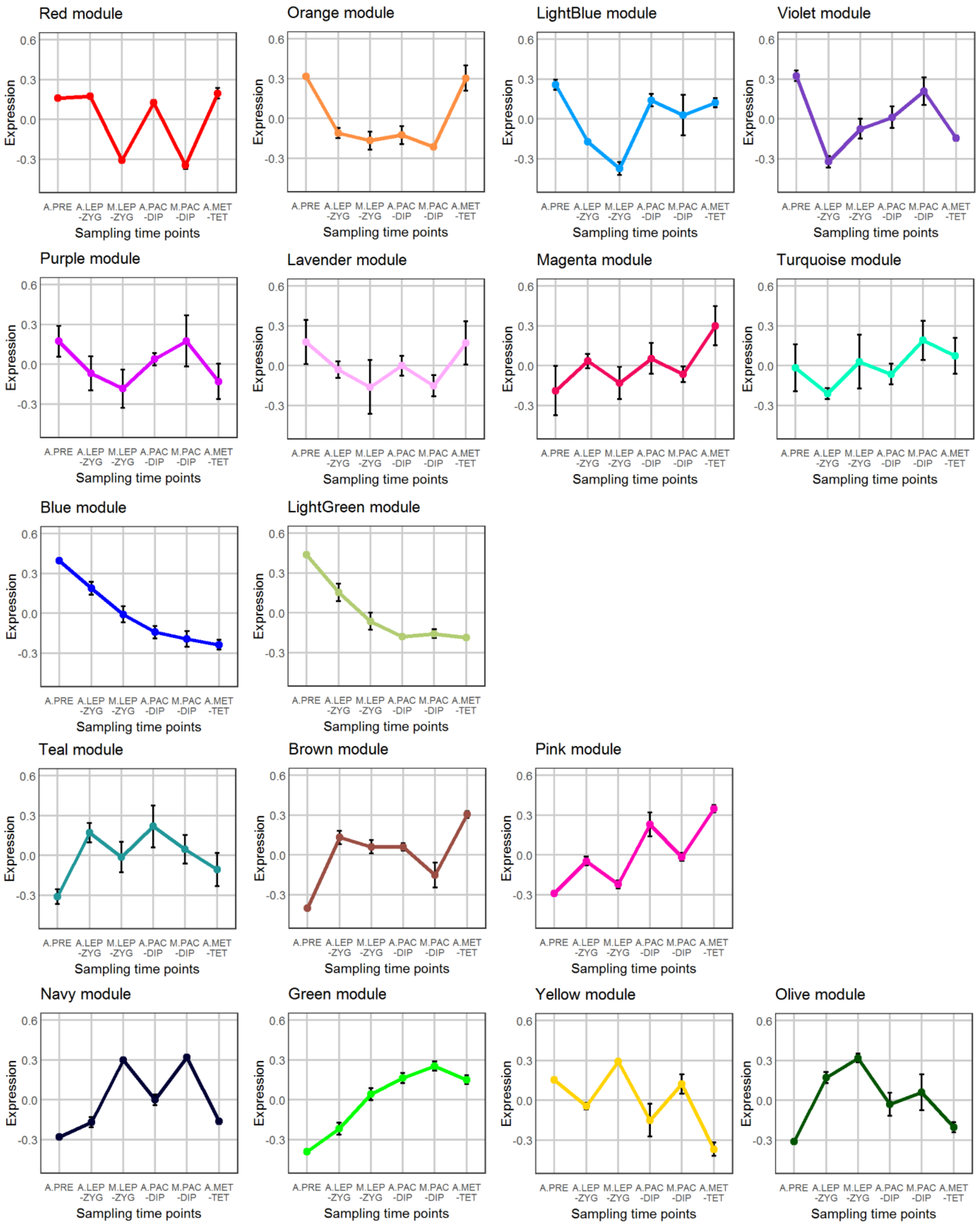

Supplementary Figure 3: WGCNA analysis of co-expressed genes. A total of 17 modules were found in anther and meiocyte transcriptomes. The samples (3 replicates each) are A.Pre, anther pre-meiosis; A.LepZyg, anther leptotene–zygotene; M.LepZyg, meiocyte leptotene–zygotene; A.PacDip,

anther pachytene–diplotene; M.PacDip, meiocyte pachytene–diplotene; A.MetTet, anther metaphase I–tetrad. The prefixes A. and M. in the sample names depict anther and meiocyte samples, respectively.

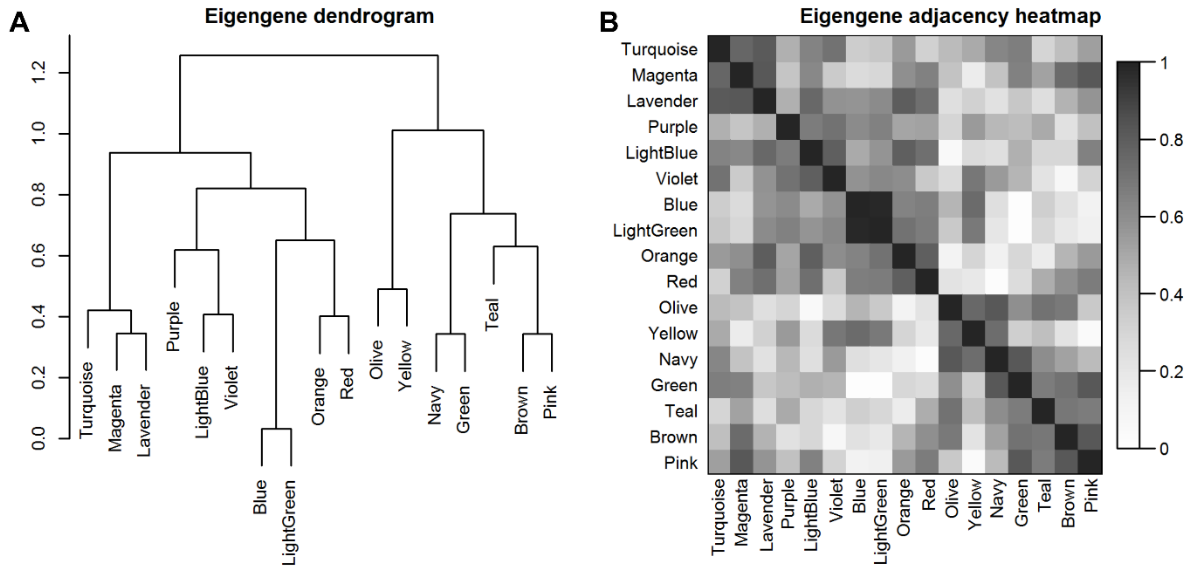

Supplementary Figure 4: Differential eigengene network analysis. **A)** Clustering dendrogram of consensus module eigengenes. **B)** Heatmap of eigengene adjacencies in barley anther and meiocyte samples. Each row and column corresponds to one eigengene labelled by consensus module colour in the band. Within the heat map black indicates high adjacency (positive correlation) and white low adjacency (no correlation) as shown by the colour legend.

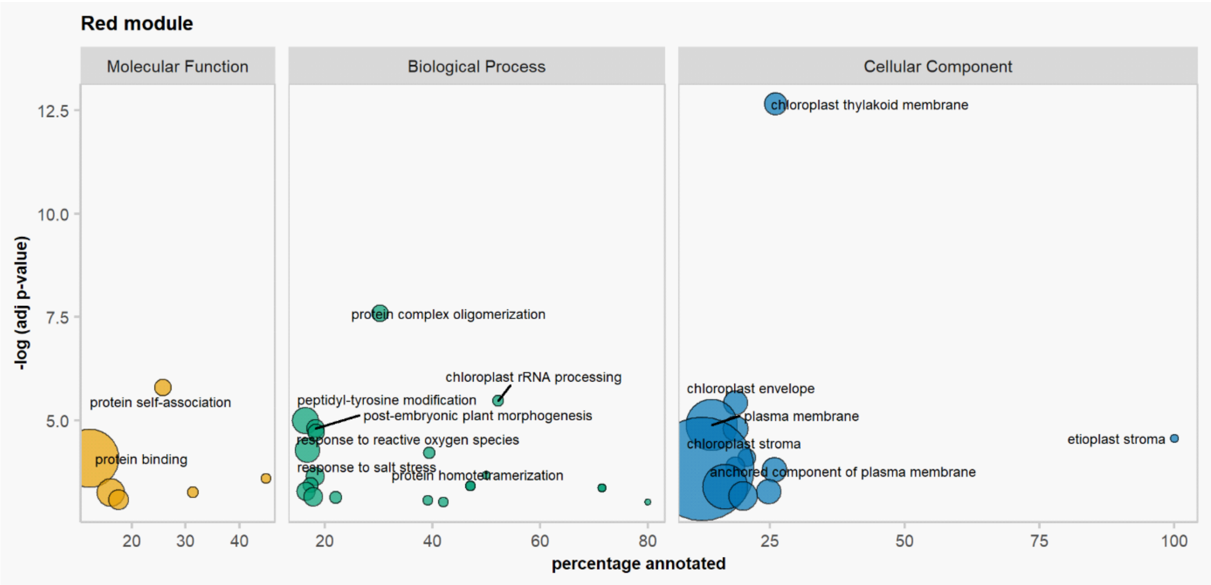

Supplementary Figure 5: Gene ontology enrichment of the red module of the WGCNA. Size of bubbles correspond to total number of proteins associated with the GO term.

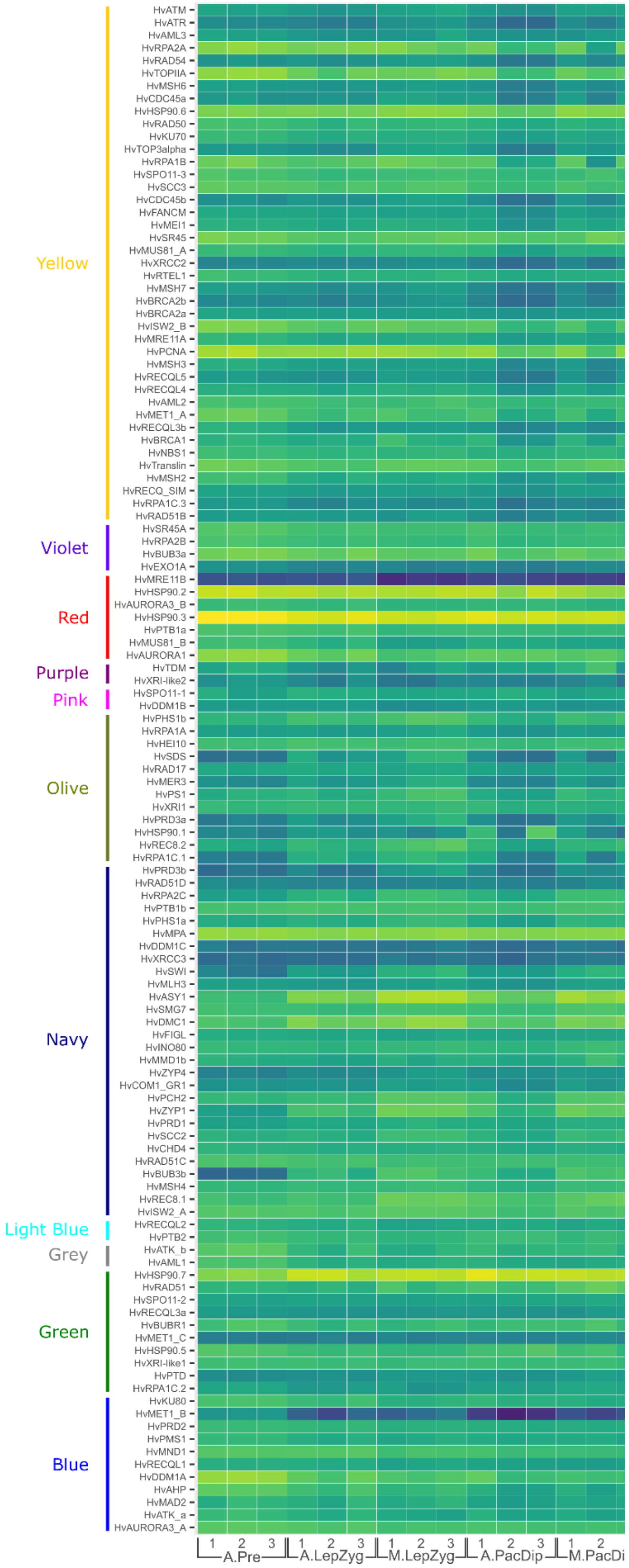

Supplementary Figure 6: Heatmap of meiotic gene expression. Only genes without a statistically significant log fold change in expression are shown. Genes are ordered and labelled by WGCNA module on the vertical axis. The samples (3 replicates each) are A.Pre, anther pre-meiosis; A.LepZyg, anther leptotene–zygotene; M.LepZyg, meiocyte leptotene–zygotene; A.PacDip, anther pachytene–diplotene; M.PacDip, meiocyte pachytene–diplotene; A.MetTet, anther metaphase I–tetrad. The prefixes A. and M. in the sample names depict anther and meiocyte samples, respectively.

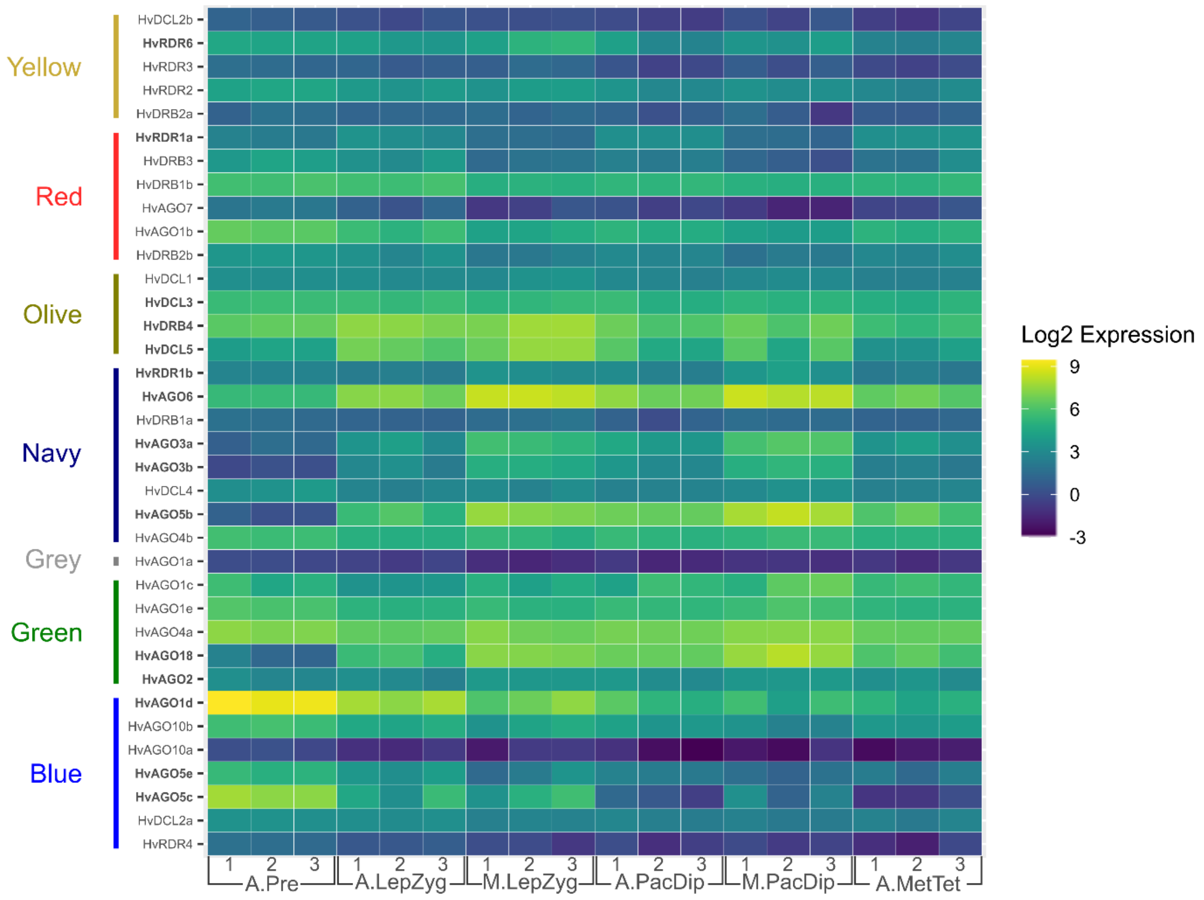

Supplementary Figure 7: A heatmap of transcript levels of genes involved in post-transcriptional regulation. Changes of expression levels of different genes involved in small RNA pathways and N 6-methyladenosine (m6A) mRNA methylation were determined by comparing meiocytes and anthers of the same developmental stage or anthers only at different stages. Transcript counts for each gene were extracted from total expression data, log transformed to normalise distribution, and plotted using ggplot2 (Wickham, 2016) in R (this study, available at www.github.com/BioJNO/BAnTr). The genes for which protein products were detected in anther proteomics (Lewandowska et al., 2019) are in bold. The samples (3 replicates each) are A.Pre, anther pre-meiosis; A.LepZyg, anther leptotene–zygotene; M.LepZyg, meiocyte leptotene–zygotene; A.PacDip, anther pachytene–diplotene; M.PacDip, meiocyte pachytene–diplotene; A.MetTet, anther metaphase I–tetrad. The prefixes A. and M. in the sample names depict anther and meiocyte samples, respectively.

3.2 Supplementary Tables

Supplementary Table 1 Raw read counts.

Sample	Replicate 1	Replicate 2	Replicate 3	Replicate 4
**Anther Premeiosis**	100915174	101362360	139765336	
**Anther Leptotene – Zygotene**	67176280	169770980	115137790	
**Anther Pachytene – Diplotene**	102527650	56910660	80108962	
**Anther Metaphase I – Tetrad**	80158780	90083264	52027680	
**Meiocyte Leptotene – Zygotene**	172172704	136784144	167233230	
**Meiocyte Pachytene – Diplotene**	175748442	182125688	154480364	
**Germinating Embryo**	59029134	66369986	63377518	56853984

Supplementary Table 2 Number of genes per WGCNA modules.

**Modules**	**Blue**	**Red**	**Green**	**Navy**	**Pink**	**Olive**
total	6209	5495	5047	3656	2996	2598
protein-coding	2660	4483	4488	3199	2511	1673
lncRNA	3135	795	406	327	396	758
unclassified	414	217	153	130	89	167
**Modules**	**Yellow**	**LightBlue**	**Brown**	**Turquoise**	**Violet**	**Teal**
total	2161	542	402	396	289	245
protein-coding	1935	450	338	361	275	197
lncRNA	174	72	43	26	10	32
unclassified	52	20	21	9	4	16
**Modules**	**Lavender**	**LightGreen**	**Magenta**	**Orange**	**Purple**	**Grey**
total	206	81	74	41	38	1442
protein-coding	174	18	65	36	36	552
lncRNA	26	58	8	5	2	816
unclassified	6	5	1	0	0	74

Supplementary Table 4: Differential expressed genes and differential expressed transcripts. Results split by the contrast groups.

Comparisons	A.LEP–ZYG vs. A.PRE	A.PAC–DIP vs. A.LEP–ZYG	A.MET–TET vs. A.PAC–DIP	M.LEP–ZYG vs. A.LEP–ZYG	M.PAC–DIP vs. A.PAC–DIP	M.PAC–DIP vs. M.LEP–ZYG
**Differential expressed genes**						
Upregulated	1929	1003	163	1713	338	2
Downregulated	4190	1131	1	3113	1678	0
**Differential expressed transcripts**						
Upregulated	1157	232	89	1615	306	2
Downregulated	4388	39	1	2510	1164	0

The comparisons are: A.LEP–ZYG vs. A.PRE, anthers at leptotene–zygotene versus anthers at pre-meiosis; A.PAC–DIP vs. A.LEP–ZYG, anthers at pachytene–diplotene versus anthers at leptotene–zygotene; A.MET–TET vs. A.PAC–DIP, anthers at metaphase I–tetrad versus anthers at pachytene–diplotene; M.LEP–ZYG vs. A.LEP–ZYG, meiocytes at leptotene–zygotene versus anthers at the same stage; and M.PAC–DIP vs. A.PAC–DIP, meiocytes at pachytene–diplotene versus anthers at the same stage. The prefixes A. and M. in the sample names depict anther and meiocyte samples, respectively.

The following tables are loaded as Excel files:

Supplementary Table 3: GO enrichment analysis results.

Supplementary Table 5: lncRNA database BLAST hits by classification.

Supplementary Table 6: Differentially Expressed E3 Ubiquitin ligases.

Supplementary Table 7: Expression of sRNA gene silencing orthologues .

Supplementary Table 8: Differential expression analysis results for Meiotic genes.

Supplementary Table 9: Distribution of transcription factor families in different WGCNA modules.

Supplementary Table 10: Transcription factor families in different comparisons.
